# Comprehensive characterization of the impairing effects of *Nosema bombycis* on the host digestive integrity and function

**DOI:** 10.1128/msphere.00095-25

**Published:** 2025-07-22

**Authors:** Lu Cao, Siying Yin, Dexu Liu, Yunlin Tang, Wenxin Yang, Maoshuang Ran, Jie Chen, Guoqing Pan, Zeyang Zhou, Jialing Bao

**Affiliations:** 1 The State Key Laboratory of Resource Insects, Southwest University 26463, Chongqing, China; 2 Chongqing Key Laboratory of Microsporidia Infection and Control, Southwest University 26463, Chongqing, China; Shenzhen Institute of Advanced Technology, Shenzhen, China

**Keywords:** *Nosema bombycis*, barrier integrity, gut microbiome, digestive capability, chitin deacetylase

## Abstract

*Nosema bombycis* (*N. bombycis*) infection causes growth retardation and indigestion of silkworms (*Bombyx mori*). The digestive tract is the primary infection route; thereby, in this study, we comprehensively investigated the disturbances of the digestive tract upon *N. bombycis* infection. Electron microscopy demonstrated the structure and integrity impairments of the peritrophic membrane and midgut after infection. A quantitative proteomic approach identified approximately 2,893 dysregulated proteins in the midgut after infection. Next, 16S rRNA and ITS analysis of the digestive juice and stool samples revealed that the gut microbiome profiles were significantly disturbed, such as bacterial genus *Acinetobacter*, *Staphylococcus,* as well as eukaryotic *Penicillium*, *Chaetasbolisia,* and so on. In addition, various digestive enzyme expressions were also disturbed upon *N. bombycis* infection. More interestingly, we found that chitin deacetylase BmCDA8, one of the top dysregulated host proteins, functioned as the key protective enzyme against *N. bombycis* since knocking down BmCDA8 leads to increased barrier permeability and higher *N. bombycis* loads. We also identified 24 *N*. *bombycis* proteins that would potentially interact with BmCDA8. Taken together, our investigation will help to fully elucidate the impairing effects of *N. bombycis* on host and to identify novel pathogen control targets.

**IMPORTANCE:**

The digestive tract is the major infection route of many pathogens. A comprehensive view of the impairing effects of *N. bombycis* on *B. mori*’s digestive tract integrity, microbiome, and enzyme compositions is greatly needed. Therefore, we applied electron microscopy, quantitative proteomics, and microbiome analysis to clearly demonstrate that *N. bombycis* infections did impair host intestinal integrity, changed the gut microbiome composition, and dysregulated digestive enzymes. Interestingly, we found that the chitin deacetylase of *B. mori* exerts an essential protective role, and *N. bombycis* effector proteins may interact directly with it to facilitate the impairing effects. Our findings provide a comprehensive view and decode the phenomena of silkworm indigestion and growth retardation after *N. bombycis* infection and will provide potential targets for disease prevention and control.

## INTRODUCTION

The microsporidia *Nosema bombycis* (*N. bombycis*) causes pébrine disease in silkworms (*B. mori*) (1, 2). The digestive tract is the major infection route of *N. bombycis*, and they live an obligatory intracellular life cycle and propagate within the host (2–4). Therefore, the digestive tract integrity and the cellular functions, as well as the related digestive capabilities, would be profoundly affected upon *N. bombycis* infection.

The *B. mori* digestive barrier consists of the peritrophic membrane and the intestinal epithelium. Chitin is the major component of the peritrophic membrane, which protects the midgut from food, ingested toxins, and infecting pathogens (5, 6). Chitin is processed by chitin deacetylases (CDAs) for better assembly of peritrophic matrices (7). Chitin deacetylase regulates the spatial arrangement and assembly patterns of chitin molecules within the peritrophic membrane through its deacetylation activity, thereby enhancing interactions between the peritrophic membrane and structural proteins (8). As a result, the alteration of BmCDA8 would affect the barrier integrity, as well as the related digestive tract contents such as microbiota and enzymes. Nevertheless, the processed chitosan is more protective against biological and pathogenic attacks (9). Various silkworm pathogens have been reported to affect host digestive barrier integrity, protein expression, and metabolism. For instance, BmNPV and BmCPV disturbed the midgut protein profiles upon infections (10, 11). *N. bombycis* modulated gene expression associated with ATP production, protein degradation, and fat metabolism (12, 13). Therefore, it will be of great interest to investigate the proteomic patterns of the silkworm midgut during *N. bombycis* infection.

The gut microbiome is also a key component of barrier function (14). Various studies revealed the disturbing effects of microsporidia infection on host gut bacterial or fungal groups. For example, one study found that *N. bombycis* infection affected the bacterial phylum of Proteobacteria, Actinobacteria, and Firmicutes in the silkworm gut, and Kyoto Encyclopedia of Genes and Genomes (KEGG) analysis revealed that the carbohydrate metabolism, amino acid metabolism, and energy metabolism pathways were dysregulated (15). Similar disturbance effects could also be found in other insect hosts such as mosquitoes, and when they were infected by microsporidia, the gut bacterium composition would change, especially the species related to metabolism, such as the pentose phosphate pathway and synthesis of antibiotics such as ansamycin and vancomycin (16). In addition to commensal prokaryotes, the eukaryotes are equally important and would also be disturbed by microsporidia infections (17, 18). The effects of *N. bombycis* infection on both gut prokaryotes and eukaryotes are much needed but have not been fully elucidated yet.

Changes in gut barrier integrity and function would definitely affect the digestive enzymes and their capabilities. Previous studies demonstrated that silkworms’ digestive proteins were susceptible to pathogen infections. For instance, BmNPV inhibited the digestive enzymes such as trypsin and serine proteases upon infection, while lipase-1 and serine protease homologs (SPHs) were shown to have prominent anti-BmNPV activity (19–21). It is of great importance to fully assess whether and how *N. bombycis* modulate silkworm digestive enzymes.

Therefore, in this study, a comprehensive investigation was carried out to explore and elucidate the undermining effects of *N. bombycis* on the host gut, including the integrity and barrier function of the midgut, the microbiome changes, as well as the digestive enzymes and their capabilities. Electron microscopy, a label-free quantitative proteomic approach, and the microorganisms’ 16S rRNA and ITS analysis would be applied in the study. Our investigations and findings will help to fully elucidate the molecular mechanisms of the dysregulation effects of *N. bombycis* on the host gut, as well as to shed light on developing novel strategies for *N. bombycis* prevention and control.

## MATERIALS AND METHODS

### *N. bombycis* spores

*N. bombycis* isolate CQ1 was isolated and preserved at the China Veterinary Culture Collection Center (No. 102059) (22). The spores were purified by centrifugation at 10,000 × *g* for 20 min in 75% Percoll (17089101, Cytiva, USA). The purified spores were washed three times with sterilized water and were ready for use.

### Insect rearing and *N. bombycis* infection

*B. mori* larvae (Dazao P50) were reared on fresh mulberry leaves in an environment of 12 h light/12 h dark, with 25°C ± 1°C and 70%–85% relative humidity.

The fifth instar stage of silkworm larvae was orally infected by *N. bombycis*. In brief, the same amount of *N. bombycis* spores (10^6^ spores/mL in distilled water) was applied onto equally cut mulberry leaves, and each silkworm was fed one leaf each time, to make sure all individuals got the same amount of *N. bombycis* infection.

The larval body weights were measured by analytical balance (HUAZHI, China), at 4 dpi for the *N. bombycis*-infected group and control groups (*n* = 15).

### Scanning electron microscopy

The midgut and peritrophic membrane of silkworms were dissected longitudinally, fixed on foam paper with insect needles, and washed with phosphate-buffered saline (PBS). Samples were then fixed in 4% paraformaldehyde. The fixed tissue materials were washed with PBS, and gradient ethanol dehydrations (80%, 90%, and 100%) were performed at room temperature. Completely dehydrated material was washed in an ethanol and xylene mixture solution (1/2 xylene) and then in 100% xylene until the tissue material was completely transparent. The processed samples were then adhered, vacuumed, sprayed with gold for about 30 s, and proceeded under electron microscopy.

A Phenom Pro benchtop scanning electron microscope (SEM; Phenom, Netherlands) was used in this experiment. The detection acceleration voltage was 4.8–20.5 kV and was continuously adjustable. The electron optical magnification was 350,000×, and the optical magnification was 27–160×. The resolution reached 6 nm.

### Peritrophic membrane permeability assay

Peritrophic membranes were dissected from silkworm larvae and rinsed with PBS. The permeability was assessed by incubating in a solution of blue dextran (20 mg/mL) (Macklin, China). After 36 h of incubation, the absorbance is determined at Abs 610 nm. For assessment of BmCDA8 effect on membrane permeability, the recombinant protein was pre-incubated (0.47 mg/mL) with the membrane for 2 h before incubation with blue dextran for permeability assay.

### Immunofluorescence assay

To observe the changes in chitin in the peritrophic membrane after *N. bombycis* infection, the sections were incubated with calcofluor white stain (Fluorescent brightener 28, FB 28) (MS4040, maokangbio, China) for 30 minutes and washed three times with PBST (0.01 M PBS + 0.05% Tween 20) each for 5 min.

To observe the changes in chitin in the peritrophic membrane and the expression of BmCDA8, the sections were incubated with mouse anti-BmCDA8 polyclonal antibody (1:200) at 37°C for 90 min and washed three times with PBST each for 5 min, followed by incubation with goat anti-mouse secondary antibody labeled with Alexa Fluor 594 (A11005, Invitrogen) for 45 min. Chitin was stained with fluorescent brightener 28 for 30 minutes. The slides were washed three times with PBST, then suspended in Antifade Mounting Medium (P0126, Beyotime) cover glass.

### LC-MS/MS analysis

The silkworm midgut was ground in liquid nitrogen, and the total proteins were extracted using lysis buffer (20 mM Tris, pH 7.4, 0.15 M NaCl, 1 mM EDTA, 0.1% Triton-X, 0.1% sodium dodecyl sulfate). The samples were centrifuged at 13,000 × *g* for 30 min, and then the supernatant was collected and trypsin processed (23). The processed protein samples were then chromatographically separated and analyzed by mass spectrometry using Q-Exactive series mass spectrometers. MaxQuant (version 1.5.3.17) software was used for database searching with the raw file for LFQ analysis, based on *B. mori* and *N. bombycis* information in the Uniprot database.

Using the Complex heat map R (R Version 3.4) to analyze protein cluster. The CELLO (http://cello.life.nctu.edu.tw/) method was used for predictive analysis of subcellular localization. Protein domain analysis was performed using the Pfam database and InterProScan software. GO category analysis was performed using Blast2GO software and KEGG path annotation using KAAS (KEGG Automatic Annotation Server) software. Fisher’s Exact Test was used for enrichment analysis. Protein interaction network analysis based on IntAct (http://www.ebi.ac.uk/intact/main.xhtml) or STRI databases and CytoScape software.

### Gut microbiota analysis

Digestive juice (4 dpi) and stool samples (5 dpi) were collected from each group. Genomic DNA was extracted using the OMEGA Soil DNA Kit (D5625-01) (Omega Bio-Tek, Norcross, GA, USA). For bacteria, the V3-V4 region of the 16S rRNA gene was amplified with the primer set 338F/806R. For fungi, the fungal ITS1 was determined using ITS5F and ITS1R primers. PCR products were detected by 2% agarose gel electrophoresis, and the target fragments were cut and recovered by the Quant-iT PicoGreen dsDNA Assay Kit. The library was constructed using TruSeq Nano DNA LT Library Prep Kit from Illumina. The constructed library is inspected by Agilent Bioanalyzer 2100 and Promega QuantiFluor. Pair-end raw sequencing reads were processed with QIIME2, then the low-quality sequences were filtered and denoised, and the chimera reads were cut off using DADA2.

### Digestive enzymes expression analysis

To detect gene (*Alpha-amylase 1, Lipase 1, Trypsin 1, Alkaline phosphatase*) expression profiles in response to *N. bombycis*, silkworm midguts were dissected for total RNA extraction using TRIzol (Invitrogen, USA) and digested with DNase I (Yeasen, China). Total RNA quality was detected by 260/280 absorbance ratio and electrophoresis. The cDNA was synthesized using reverse transcriptase (Roche, Switzerland). The *B. mori* housekeeping gene actin A3 was used as an internal control for normalization. Quantitative real-time PCR was performed with a real-time PCR system (Roche). Primers are in Table S1.

### Digestive enzyme activities

For preparation of enzyme solution in digestion juice, after the hemolymph of the silkworm samples were taken, the complete gut was taken, placed in a centrifuge tube, and centrifuged at 12,000 rpm for 15 min at 4°C; centrifugation was repeated twice; and the supernatant was taken as the enzyme solution to be tested. Protein concentration was determined with the BCA Protein Assay Kit (P0012, Beyotime). Enzyme activities were determined by α-Amylase Assay Kit (C016-1-2), Lipase assay kit (A054-1-1), Trypsin assay kit (A080-2-1), and Alkaline phosphatase assay kit (A059-1-1) (NanJing Jiancheng Bioengineering Institute, China).

### Recombinant BmCDA8 purification and enzyme activity assay

The coding region of *B. mori* chitin deacetylase 8 (XP_004923455.1) was PCR-amplified from *B. mori* cDNA with specific primers (Table S1) and cloned into the vector pCold-TF. The recombinant plasmids were transformed into *E. coli* Rosetta (CD801-02, TransGen Biotech, China) and cultured in Luria-Bertani medium at 37°C to an OD 600 of 0.6. Recombinant protein expression was induced by adding 0.5 mM isopropyl β-d-thiogalactopyranoside for 22 h at 16°C. Proteins were purified using a Ni^2+^-nitrilotriacetic acid column (30210, QIAGEN, Germany) according to the manufacturer’s instructions. Coomassie blue staining was applied to confirm the purification of BmCDA8. To assess the enzyme activity of recombinant BmCDA8, 4-nitroacetanilide (N838405, Macklin, Shanghai, China) was used as substrate to generate 4-nitroaniline (N814819, Macklin, Shanghai, China) under the catalysis of chitin deacetylase, and the absorbance value of the reaction product was measured at 405 nm.

### RNA interference assay

The dsRNAs targeting 250 bp regions of BmCDA8 and 240 bp regions of enhanced green fluorescent protein (EGFP) genes were synthesized by specific primers (Table S1). The T7 RiboMAX Express RNAi System (P1700, Promega, USA) was applied according to the manufacturer’s instructions. Purified dsRNAs were assessed by gel electrophoresis (Fig. S1). The acquisition, injection dose, and injection process of 5 µg of dsRNA-*BmCDA8* were performed as described (24). An equal volume of dsRNA-*EGFP* was simultaneously injected as a control. The success of RNA interference was confirmed by Western blot. Total proteins were separated using 10% SDS-PAGE and transferred to a PVDF membrane (Millipore, USA). The membrane was incubated with anti-BmCDA8 (1:800) for 1 h at 37°C. Thereafter, it was washed three times with TBST and incubated for 1 h at 37°C with HRP-linked anti-mouse IgG antibody (BL001A, Bioshap, China). After three washes with TBST, the membrane was exposed to an ECL western blot detection kit (BG0001, BIOGROUND, China) and imaged using Western chemical exposure system (Clinx, China).

After RNAi knockdown of BmCDA8 expression, an analytical balance was used to measure the larval body weight for the *N. bombycis* + dsRNA-CDA8 group, *N. bombycis*-infected group, and control group (*n* = 15). In addition, the gene expression changes of digestive enzymes were also measured. PCR analysis was conducted to evaluate alterations in representative gut microbiota species (*Enterococcus faecalis* strain YM0831 and *Bacillus subtilis* strain SEM-2), with genomic DNA standardized to 50 ng per sample.

### Detection of *N. bombycis* load/proliferations within the host

To determine the *N. bombycis* loads, genomic DNA was extracted from the samples using the EZNA Tissue DNA Kit (OMEGA, USA). *N. bombycis* β-tubulin (EOB14994.2) was detected by qPCR assay using specific primers (Table S1), and the pathogen copy numbers were calculated. The recombinant pMD 19-T Vector plasmid (1.8 × 10^8^ copies; 6013, TaKaRa) integrated with *N. bombycis* β-tubulin was used as the standard.

### His pull-down

The full-length BmCDA8 was amplified and cloned into the pCold-TF vectors. This His-tagged recombinant BmCDA8 protein was incubated with the total proteins extracted from *N. bombycis*. Directly interacting proteins were pulled down using the Dynabeads and His-Tag Isolation & Pulldown kit (10103D; Invitrogen), following the manufacturer’s instructions.

### Yeast two-hybrid assay

BmCDA8 and vacuolar protein sorting-associated protein 9a (NbVPS9a) and GCN1-like translational activator (NbGCN1) were amplified and cloned into pGBKT7 or pGADT7 plasmids (primers are in Table S1). The bait and prey plasmids were co-transformed with the yeast strain Y2HGold (MF2351, MKBio, China). The positive control pGBKT7-53/pGADT7-T and negative control pGBKT7-Lam/pGADT7-T were transformed in the same manner. The cultured strains were placed on an SD/-Ade/-His/-Leu/-Trp with X-α-gal (QDO) solid culture plate for screening.

### Statistical analysis

Statistical differences were evaluated using Student’s t-test. The level of statistical significance was set at *P < 0.05, **= < 0.01, ***P < 0.001, ****P < 0.0001.

## RESULTS

### *N. bombycis* impaired silkworm digestive tract structures and permeability

**Fig 1:**
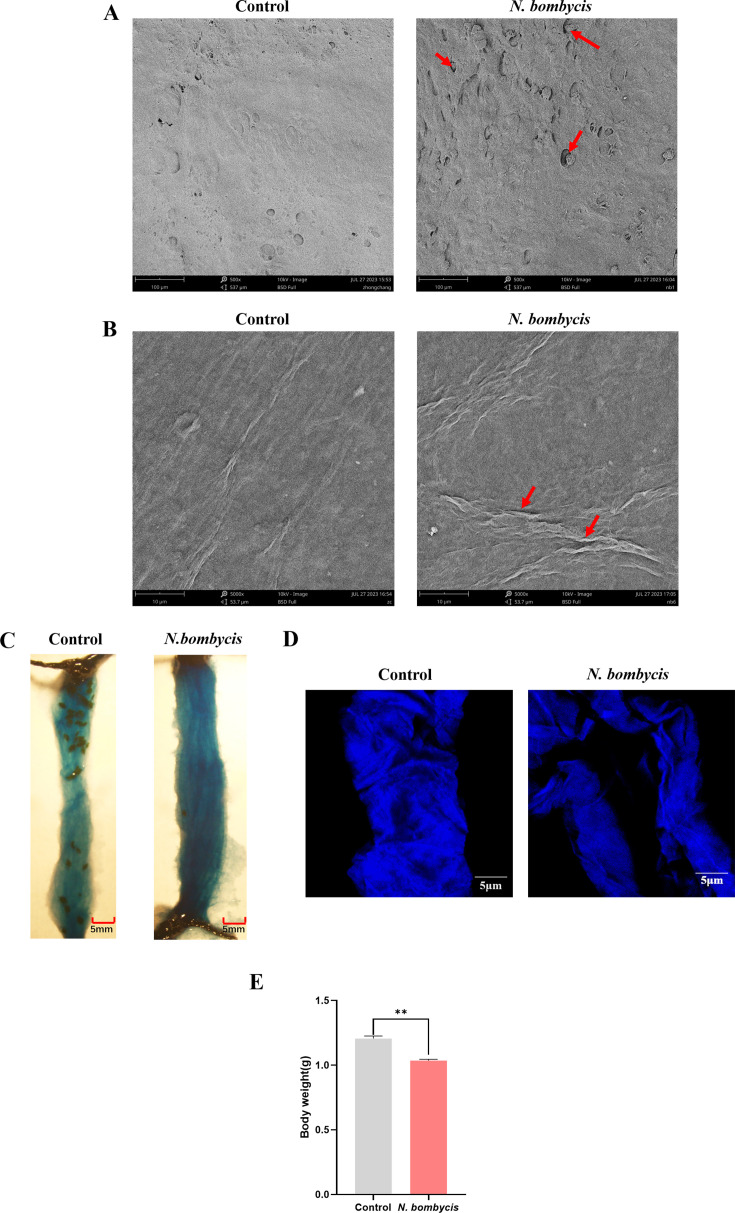
Digestive barrier impairments after *N. bombycis* infection (4 dpi). (**A**) SEM observation of midgut membrane. More penetrations and holes (red arrows) after *N. bombycis* infection (magnification, 500×). (**B**) SEM observation of the peritrophic membrane. Significantly more anomalous folds (red arrows) after *N. bombycis* infection (magnification, 5,000×). (**C**) The peritrophic membrane permeability was assessed by the accumulation of blue dextran. The amount of blue dextran increased in the peritrophic membrane of the *N. bombycis* group. (**D**) Peritrophic membrane was stained with calcofluor white (Fluorescent brightener 28). The peritrophic membrane of the *N. bombycis*-infected group exhibited more distortion (scale bar: 5 µm). (**E**) Body weight measurement after *N. bombycis* infection. The body weight of *N. bombycis*-infected group was significantly lower than the control group (*n* = 15, **P < 0.05).

The structure of the silkworm peritrophic membrane and midgut membrane was first assessed by SEM. Results showed that *N. bombycis* infection caused anomalous folds, penetrations, and holes on the surfaces of both silkworm midgut and peritrophic membrane (Fig. 1A and B). Next, the integrity and permeability of the peritrophic membrane were assessed. Absorbance quantifications revealed that significantly more amount of blue-color dextran passed through the membrane into the lumen of the *N. bombycis*-infected silkworms (Fig. 1C), and a more distorted structure of the peritrophic membrane was observed (Fig. 1D). As a result, the infected silkworms showed significant body weight loss compared to uninfected controls (Fig. 1E). These findings confirmed the impairing effects of *N. bombycis* infections on host digestive tract integrity.

### *N. bombycis* infection altered silkworm mid-gut protein expressions

Label-free quantitative proteome analysis was applied to the total proteins isolated from *N. bombycis*-infected silkworm midgut or uninfected controls. The analysis identified 2,893 upregulated/downregulated expressed proteins. Among them, the most dysregulated 114 proteins were identified based on the cutoff of twofold change and a *P*-value < 0.05. In detail, 21 proteins were listed as the most upregulated ones (Table 1), 22 proteins were the most downregulated ones (Table 2), 59 proteins appeared only after *N. bombycis* infection (Table 3), while 12 proteins disappeared after infection (Table 4).

**TABLE 1:** Upregulated proteins identified in the silkworm midgut after *N. bombycis* infection

Protein	Accession number	Molecular weight (kDa)
Insect intestinal mucin	G1FU07	31.783
Regucalcin	A0A8R2APY2	33.731
PUA domain-containing protein	A0A8R1WJB0	20.453
Ribosomal protein S27A	Q9XXZ5	17.847
V-type proton ATPase proteolipid subunit	Q1HQ99	15.841
Sr protein	Q2F654	18.553
UDP-glucose 4-epimerase	A0A8R2M494	40.279
Uncharacterized protein	A0A8R2GDC6	26.288
Rbp1-like RNA-binding protein PC	B9VSZ7	10.627
Chitin deacetylase 8	H9JW43	43.31
Collagen type IV alpha-3-binding protein	A0A8R2AK41	62.537
Kinase	A0A8R2R915	49.638
RNA helicase	Q1HQ29	59.762
Uncharacterized protein	A0A8R2AK85	25.932
Lipase domain-containing protein	A0A8R2LZP7	32.051
Purine nucleoside phosphorylase	A0A8R2ATW6	36.274
Actin-4	S5MNK8	41.822
Nucleic acid-binding protein asmtl	A0A8R2AKM8	22.677
Microtubule-associated protein Jupiter	A0A8R2LVA3	13.657
LRP16 protein	Q1HPZ5 Q1HPZ5	30.64
Gelsolin-like domain-containing protein	A0A8R1WGR3	84.261

**TABLE 2:** Downregulated proteins identified in the silkworm midgut after *N. bombycis* infection

Protein	Accession number	Molecular weight (kDa)
Aminopeptidase	I3VR78	106.31
Antennal-binding protein	Q2F5L4	15.49
Cuticle protein	A0A8R2MAB1	12.456
Alkaline phosphatase	B2ZZX0	60.263
Uncharacterized protein 19G1P (fragment)	D4QGC0	27.343
Major facilitator superfamily (MFS) profile domain-containing protein	A0A8R2GD17	54.312
Resistance to inhibitors of cholinesterase protein 3 N-terminal domain-containing protein	A0A8R2M3L8	77.273
Membrane-bound alkaline phosphatase	P29523	60.28
Uncharacterized protein	A0A8R2AR03	26.872
Cuticle protein	A0A8R1WLL5	26.998
EF-hand domain-containing protein	A0A8R2QW80	24.889
Putative cuticle protein	C0H6N4	17.064
Immulectin	Q7Z1E5	34.511
30K protein 10	H9B443	29.734
Arylphorin	Q1HPP4	83.451
Serine protease	Q58I79	29.646
CP8	Q6TUD0	9.6335
Silkworm storage protein	H9JHM9	82.807
Ubiquitin-60S ribosomal protein L40	Q9XXZ4	14.796
Twitchin	A0A8R2MBJ4	520.57
RNA-binding protein NOB1	A0A8R2G7L7	49.582
Sex-specific storage-protein 1	P09179	87.241

**TABLE 3:** Proteins appeared in the silkworm midgut after infection *N. bombycis* infection

Protein	Accession number	Molecular weight (kDa)
Fibroin light chain (fragment)	Q9BIF8	24.676
NADP-dependent oxidoreductase domain-containing protein	A0A8R2M1L3	36.93
Transcription intermediary factor 1-alpha	A0A7R7G212	104.23
Death domain-containing protein	A0A8R2AFR7	45.085
Chitin-binding type-4 domain-containing protein	A0A8R1WG84	25.022
Macrophage erythroblast attacher	A0A8R1WGQ1	44.929
EF-hand domain-containing protein	A0A8R1WHS3	27.993
EF-hand domain-containing protein	A0A8R1WK95	22.85
SWIM-type domain-containing protein	A0A8R2GAV4	75.626
Uncharacterized protein	A0A8R2DN62	97.249
UPF0047 protein yjbQ	A0A8R1WL46	18.179
WD repeat-containing protein 55 homolog	A0A8R1WLE7	37.051
Cysteine and histidine-rich domain-containing protein	A0A8R1WLS4	39.252
Methyltransferase-like 26	A0A8R1WMK5	25.488
Uncharacterized protein	A0A8R1WN93	27.117
Hexosyltransferase	A0A8R1WPW9	82.816
Bleomycin hydrolase	A0A8R2AIQ6	53.376
Late endosomal/lysosomal adaptor and MAPK and MTOR activator 5	A0A8R2AK28	9.6352
Regulator of nonsense transcripts 1	A0A8R2AKE5	115.6
Protein-lysine N-methyltransferase 101736309	A0A8R2AL33	24.33
Elongation factor 1-alpha	A0A8R2AL57	50.24
Ommochrome-binding protein-like	A0A8R2AL74	31.469
Peptidase S1 domain-containing protein	A0A8R2ALL2	27.371
Phosphorylase b kinase regulatory subunit	A0A8R2GA37	137.22
Pinin/SDK/MemA protein domain-containing protein	A0A8R2ALQ7	45.912
Uncharacterized protein	A0A8R2ANF6	24.252
Ataxin-10	A0A8R2ANW1	88.611
ATP synthase mitochondrial F1 complex assembly factor 2	A0A8R2AP59	31.357
RUN and FYVE domain-containing protein 2	A0A8R2C845	65.551
RNA helicase	A0A8R2AQP5	74.045
Probable cytosolic iron-sulfur protein assembly protein Ciao1	A0A8R2ASF1	37.844
Uncharacterized protein	A0A8R2AT36	12.091
Extracellular serine/threonine protein kinase four-jointed	A0A8R2C5U6	56.94
ubiquitinyl hydrolase 1	A0A8R2C9V6	177.94
GRAM domain-containing protein	A0A8R2DLU6	44.848
WH1 domain-containing protein	A0A8R2HQ30	54.445
Rab-GAP TBC domain-containing protein	A0A8R2GDM7	128.2
Cuticle protein	A0A8R2M7E1	34.902
Fasciclin-2	A0A8R2GAA3	84.371
Pre-mRNA-splicing factor RBM22	A0A8R2G9X5	46.215
Chymotrypsin-like proteinase	H9BVM5	29.781
Cuticle protein	A0A8R2QYY9	31.021
TFIIS N-terminal domain-containing protein	A0A8R2HRY9	17.533
Hyccin	A0A8R2LYA4	55.139
Lysosomal-trafficking regulator	A0A8R2LZW4	376.88
Chitin-binding type-2 domain-containing protein	A0A8R2M1X5	717.79
TIP41-like protein	A0A8R2M0A0	32.029
Uncharacterized protein	A0A8R2QYW8	73.889
CCHC-type domain-containing protein	A0A8R2M6K9	135.23
LysM domain-containing protein	A0A8R2M743	26.446
Phosphofurin acidic cluster sorting protein 1	A0A8R2M8Y3	94.933
Beta-1,3-glucanase	C8C9T4	42.009
DNA topoisomerase I	A0A8R2RBM9	70.904
Tachykinin	B3IUD3	29.042
UDP-glucuronosyltransferase	G9LPS2	58.81
Activator of basal transcription 1	Q1HDZ9 Q1HDZ9	23.878
Defective in cullin neddylation protein	Q2F653	30.582
60S ribosomal protein L35	Q5UAQ4	14.461
Actin-4	S5M0T2	41.78

**TABLE 4:** Proteins disappeared in the silkworm midgut after *N. bombycis* infection

Protein	Accession number	Molecular weight (kDa)
Adenosine deaminase (fragment)	A5HMG4	62.337
Uncharacterized protein	A0A8R1WFY7	15.993
Myrosinase 1-like	A0A8R1WGR7	56.61
Transcription initiation factor TFIID subunit 10	A0A8R1WMN0	14.37
Alpha-methylacyl-CoA racemase	A0A8R2AKG7	42.078
Rab-GAP TBC domain-containing protein	A0A8R2DM61	90.021
WH1 domain-containing protein	A0A8R2C9K7	41.105
Proline dehydrogenase	A0A8R2CA14	66.712
Uncharacterized protein	A0A8R2HMQ7	44.291
EB domain-containing protein	A0A8R2M0X2	34.568
PUM-HD domain-containing protein	A0A8R2M2I0	105.49
Trafficking protein particle complex 6b	Q1HPK2	18.612

**Fig 2:**
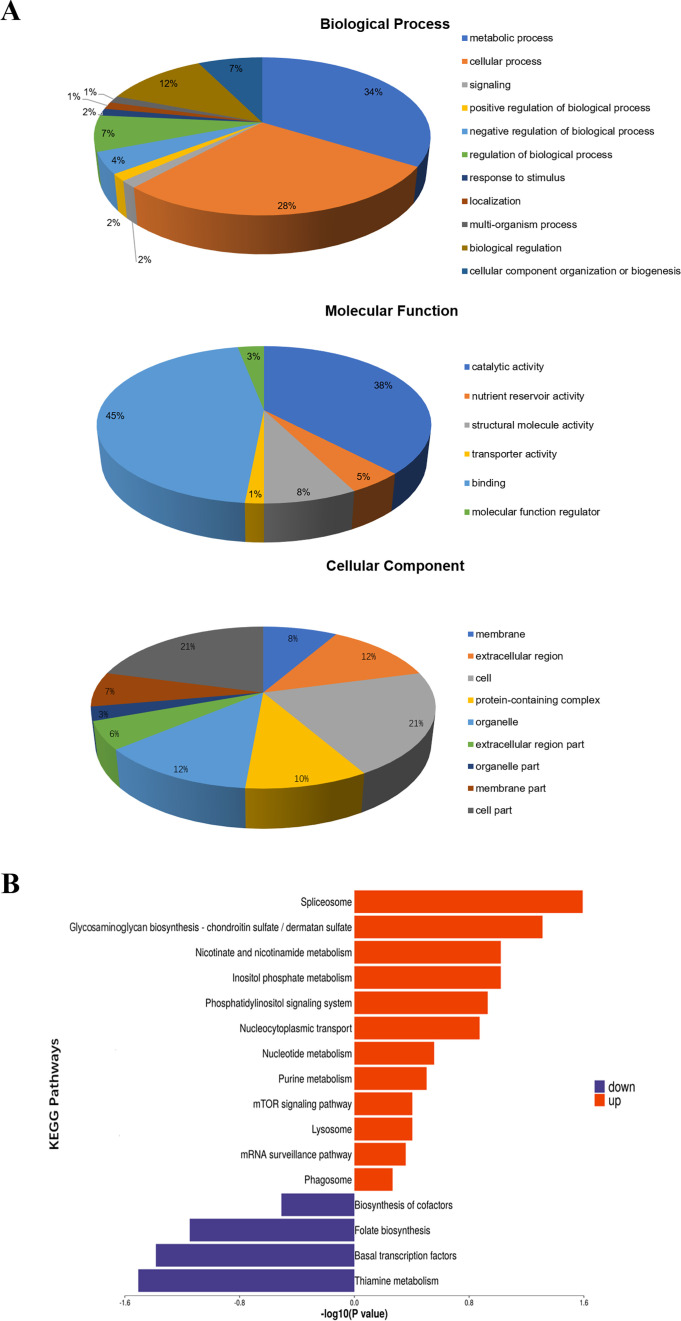
Gene Ontology analysis and KEGG pathway analysis. (**A**) The 114 *B. mori* proteins differentially expressed after *N. bombycis* infection were annotated according to biological process, cellular component, and molecular function categories. In BP, the differentially expressed proteins were enriched in processes such as metabolic, cellular functions, signaling, response to stimulus, etc. For the MF category, proteins were enriched in binding activity, catalytic activity, structural molecular activity, etc. For CC, the proteins were enriched in the cell, membrane, extracellular region, protein-containing complex, and so on. (**B**) KEGG functional analysis and the top enriched pathways analysis showed that the upregulated proteins were categorized in processes associated with spliceosome, glycosaminoglycan biosynthesis, nicotinate and nicotinamide metabolism, inositol phosphate metabolism, phosphatidylinositol signaling system, and nucleocytoplasmic transport. The downregulated proteins were categorized into cofactor biosynthesis, folate biosynthesis, basal transcription factors, and thiamine metabolism.

More importantly, we identified 36 *N*. *bombycis* proteins in the silkworm midgut samples, indicating the potential pathogenic effectors (Table 5). Next, we performed Gene Ontology (GO) analysis of the differentially expressed *B. mori* proteins. Results demonstrated that the differentially expressed proteins were enriched under the three categories: biological process (BP), cellular component (CC), and molecular function (MF). In the BP category, 34% of the proteins were enriched in the metabolic processes, followed by cellular processes (28%) and biological regulation (12%). In the MF category, 45% of the proteins were enriched in binding activity, followed by catalytic activity (38%) and structural molecular activity (8%). In the CC category, proteins were enriched in the cell (21%), membrane (12%), and extracellular region (12%) (Fig. 2A). These findings emphasized the fierce pathogen-host interactions and the profound regulating effects of infection on host cells. The KEGG functional analysis and the top enriched pathways are shown in Fig. 2B. The top upregulated proteins were categorized in processes associated with spliceosome, glycosaminoglycan biosynthesis, nicotinate and nicotinamide metabolism, inositol phosphate metabolism, phosphatidylinositol signaling system, and nucleocytoplasmic transport. For the downregulated proteins, KEGG pathways fall into cofactors biosynthesis, folate biosynthesis, basal transcription factors, and thiamine metabolism.

**TABLE 5:** *N.bombycis* proteins identified in the silkworm midgut after *N. bombycis* infection

Protein	Accession number	Molecular weight (kDa)
Protein translocation protein SEC63	R0KS42	66.285
Calcium-binding EF-hand	R0KUL6	22.755
Phospholipid/glycerol acyltransferase domain-containing protein	R0MK00	27.06
14-3-3 domain-containing protein	R0KXN9	30.135
Rnase ph	R0MDN6	20.119
Developmentally regulated GTP-binding protein 1	R0MLQ5	40.461
Fidgetin-like protein 1	R0MEU3	49.056
Actin (fragment)	R0KN67	18.109
Ubiquitin (fragment)	R0KPY3	15.818
Ras-related protein Rab-1B	R0MLC0	23.606
Tyrosine-tRNA ligase (fragment)	R0MN02	24.177
26S protease regulatory subunit 4 (fragment)	R0KRR6	26.424
Homologous-pairing protein 2 winged helix domain-containing protein	R0MI60	38.763
26S proteasome non-ATPase regulatory subunit 14 (fragment)	R0MK66	48.007
Iron-sulfur cluster assembly enzyme ISCU, mitochondrial	R0M3U3	15.387
Integrator complex subunit 11	R0M5V3	56.881
Signal peptidase-like protein	R0KW94	19.962
Uncharacterized protein	R0MIA1	20.212
26S protease regulatory subunit 6B (fragment)	R0MRG0	42.749
DNA replication licensing factor MCM6 (fragment)	R0MIE3	66.78
Ras-related protein RABA1c	R0MLD0	24.349
Asparaginyl-tRNA synthetase, cytoplasmic	R0M4Y2	36.576
Cyclin-dependent protein kinase PHO85	R0MFH2	30.638
Uncharacterized protein	R0KMZ3	34.803
Uncharacterized protein	R0M400	14.973
DNA mismatch repair protein mutS	R0KPS4	59.18
Tubulin alpha chain	R0MMA4	49.206
Glucose-6-phosphate isomerase	R0MM51	58.234
N (6)-L-threonylcarbamoyladenine synthase	R0KLP4	36.66
Uncharacterized protein	R0MHN5	6.5228
DNA primase large subunit	R0KV59	45.489
Uncharacterized protein	R0KRL7	10.055
Chromatin structure modulator	R0KLD0	11.254
Uncharacterized protein	R0KPW9	25.402
Uncharacterized protein	R0MEF6	25.677
Vacuolar proton pump subunit B	R0M3X7	54.092

### *N. bombycis* infection disturbed the silkworm gut microbiota

**Fig 3:**
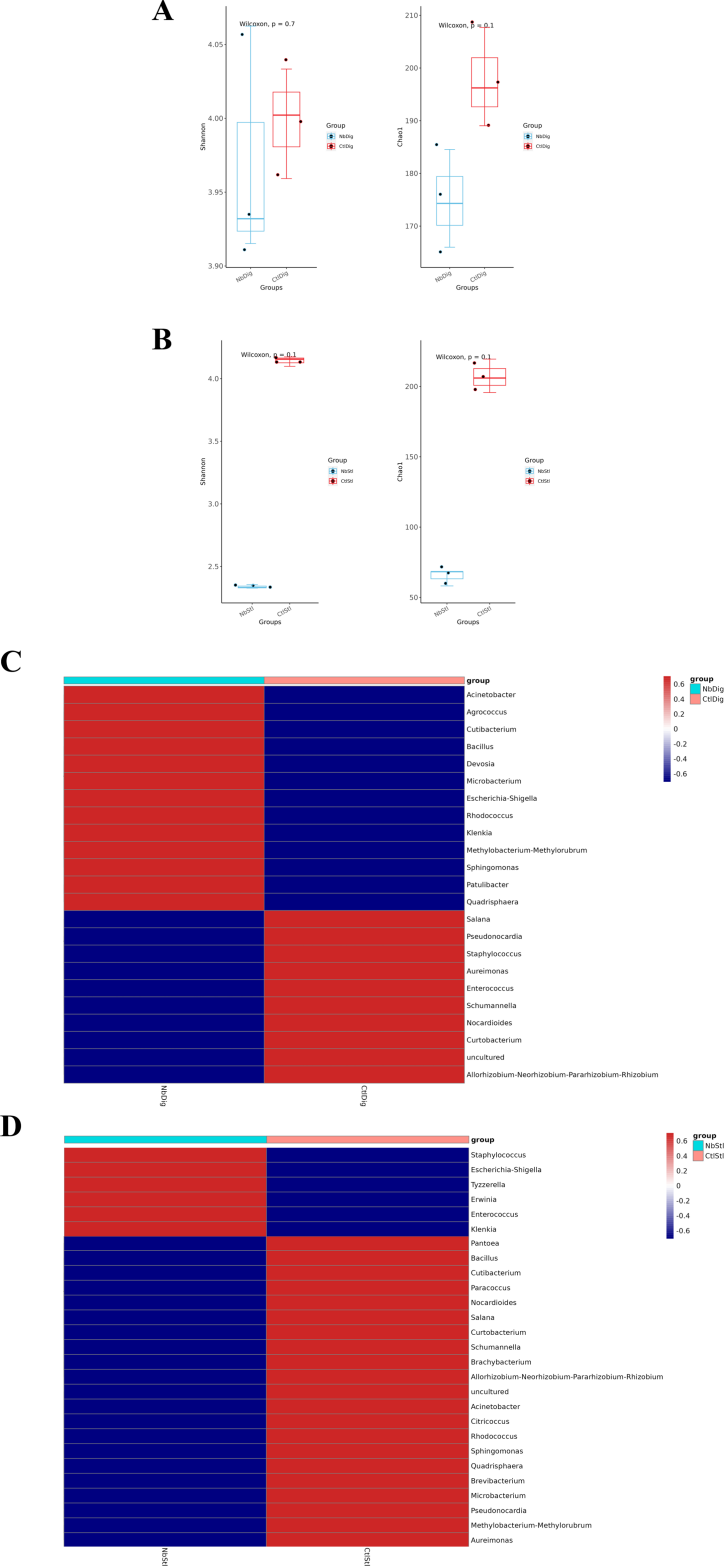
*N. bombycis* infection alters the composition of the gut bacterial communities in silkworms. (**A**) Alpha diversity analysis of digestive juice samples. The Shannon and Chao1 indices of the *N. bombycis*-infected group were lower than the control group, but not significantly. (**B**) Alpha diversity analysis of stool samples. The Shannon and Chao1 indices of the *N. bombycis*-infected group were lower than the control group, but not significantly. (**C**) Changes in bacterial composition on the genus level in digestive juice samples (NbDig = *N. bombycis*-infected digestive juice sample; CtlDig = Control digestive juice sample). (**D**) Changes in bacterial composition on the genus level in stool samples (NbStl = *N. bombycis*-infected stool sample; CtlStl = Control stool sample).

Silkworm gut prokaryotes in digestive juice and stool samples were first assessed by 16S RNA sequencing analysis, respectively. The biological diversity (Shannon or Chao indices) analysis showed that *N. bombycis* infection resulted in decreased gut microbial richness and diversity in both digestive juice and stool samples (Fig. 3A and B). Next, the composition of bacterial community analysis in the digestive juices demonstrated that *N. bombycis* infection led to the upregulation of 13 genera of microorganisms, including *Acinetobacter*, *Cutibacterium*, *Bacillus*, and *Rhodococcus,* etc.; while downregulation of 10 genera such as *Staphylococcus* and *Enterococcus* (Fig. 3C); while the *N. bombycis* infection caused upregulation of 6 genera in stool samples such as *Staphylococcus* and *Escherichia-Shigella*, and downregulation of 21 genera, such as *Pantoea*, *Cutibacterium*, and *Bacillus* (Fig. 3D). These dysregulated microorganisms were functionally associated with commensal bacteria colonization, immunological responses, digestion of polysaccharides, and nutrition uptake, etc., indicating the profound disturbance on gut bacteria caused by *N. bombycis*.

**Fig 4:**
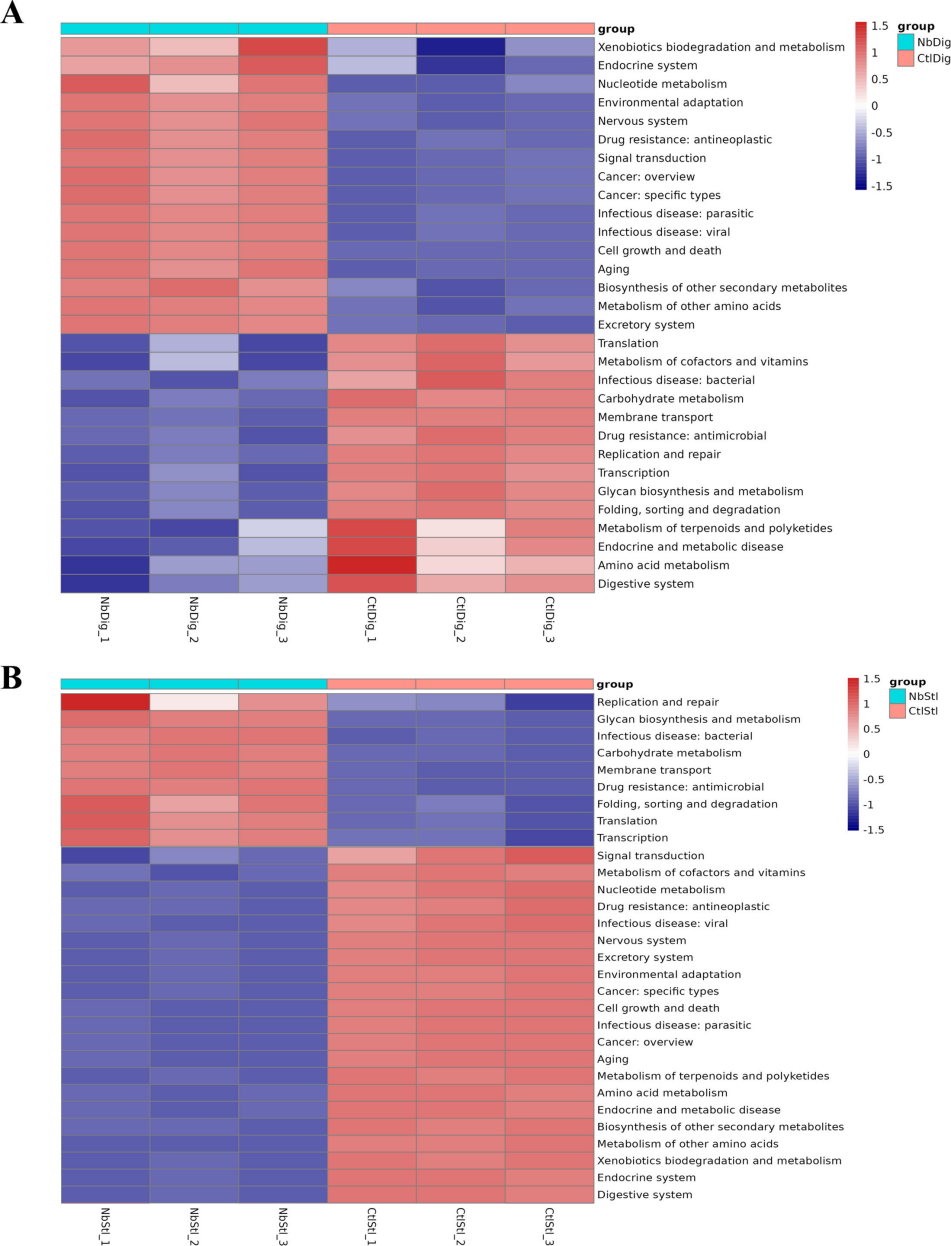
Functional analysis of gut bacterial communities after *N. bombycis* infection. (**A**) Functional KEGG pathways in digestive juice samples (three individual *N. bombycis*-infected samples: NbDig_1, NbDig_2, and NbDig_3; three individual control samples: CtlDig_1, CtlDig_2, and CtlDig_3). (**B**) Functional KEGG pathways in stool samples (three individual *N. bombycis*-infected samples: NbStl_1, NbStl _2, and NbStl _3; three individual control samples: CtlStl _1, CtlStl_2, and CtlStl_3). (*n* = 5 individual silkworms, samples were combined and analyses were done in triplicate).

KEGG pathway analysis of the digestive juice samples demonstrated the bacterial genes associated with xenobiotics biodegradation and metabolism, endocrine system, and nucleotide metabolism were upregulated, and digestive system and amino acid metabolism were downregulated (Fig. 4A). The enriched pathways analysis of the stool samples showed that microorganism genes associated with replication and repair, glycan biosynthesis and metabolism, and infectious diseases were upregulated, while pathways of digestive system, endocrine system, and xenobiotics biodegradation and metabolism were downregulated (Fig. 4B).

**Fig 5:**
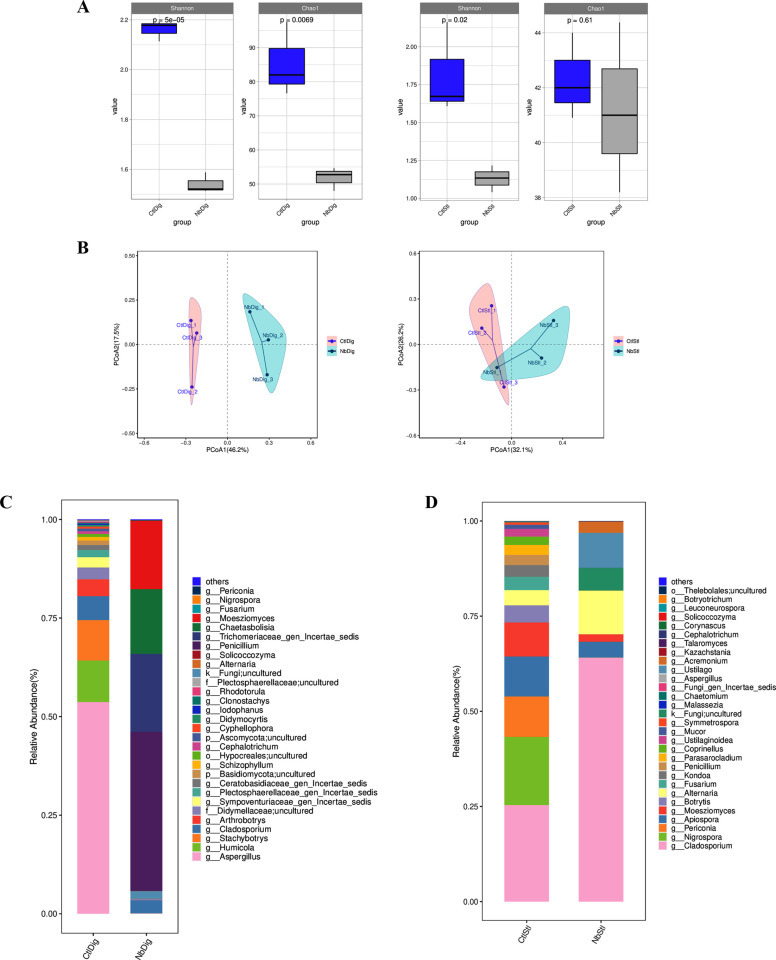
*N. bombycis* infection alters the composition of the gut fungal communities in silkworms. (**A**) Alpha diversity analysis of digestive juice and stool samples was investigated. The Shannon index of *N. bombycis*-infected group was significantly lower than the uninfected group (*P* < 0.05). In the digestive juice samples, the Chao1 index of *N. bombycis*-infected group was significantly lower than the uninfected group (*P* < 0.05). In the stool samples, the Chao1 index of *N. bombycis*-infected group was lower than uninfected group, but not significant. (**B**) Beta diversity of digestive juice and stool samples. PCoA plot showed the separation in community structure between *N. bombycis*-infected group and the uninfected group. (**C**) Relative abundance of fungal genus in digestive juice samples (NbDig = *N. bombycis*-infected digestive juice sample; CtlDig = Control digestive juice sample). (**D**) Relative abundance of fungal genus in stool samples (NbStl = *N. bombycis*-infected stool sample; CtlStl = Control stool sample).

**Fig 6:**
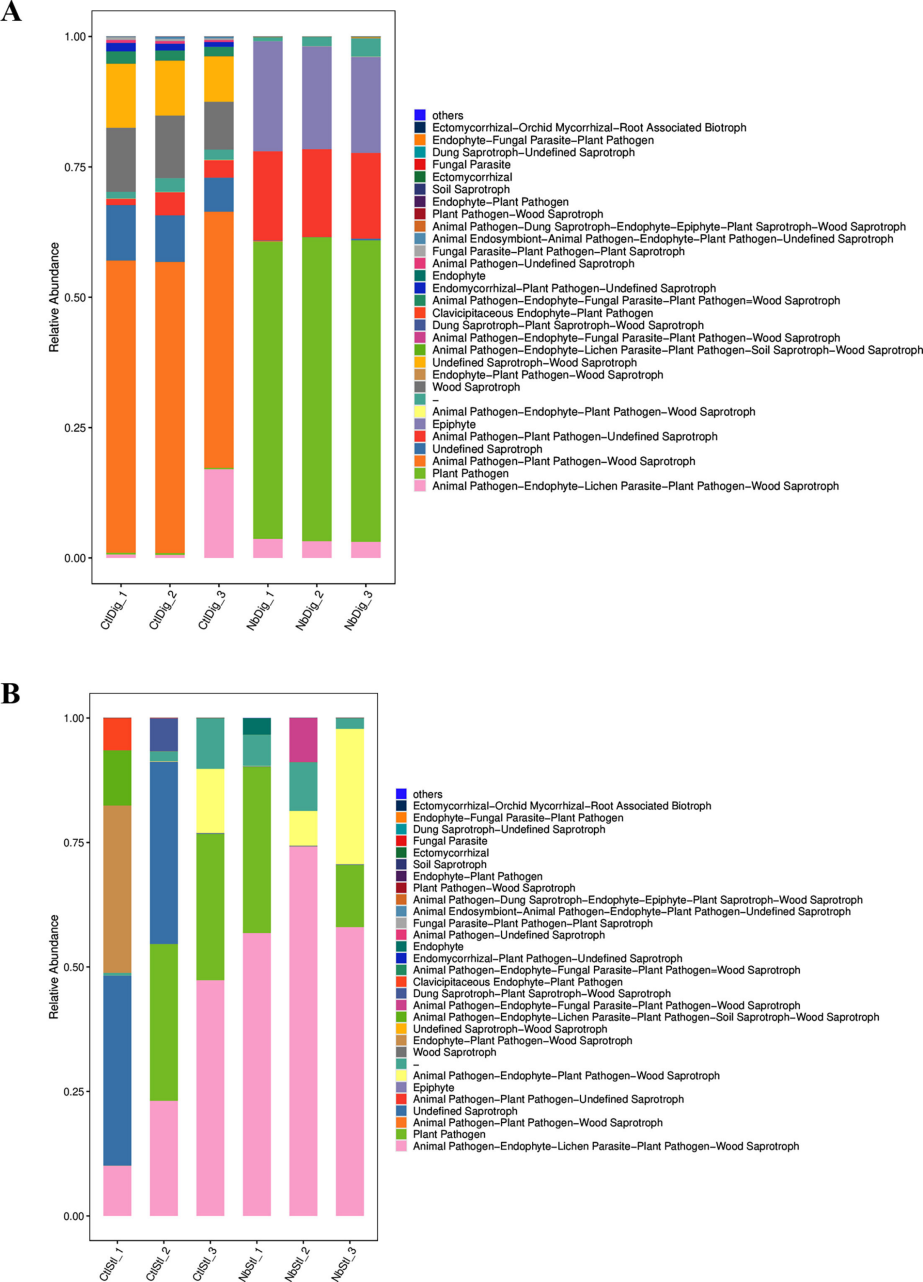
Funguild types analysis of gut fungal communities after *N. bombycis* infection. (**A**) Relative abundance of funguild types in digestive juice samples (*N. bombycis*-infected groups: NbDig_1, NbDig_2, and NbDig_3; uninfected groups: CtlDig_1, CtlDig_2, and CtlDig_3). (**B**) Relative abundance of funguild types in stool samples (*N. bombycis*-infected groups: NbStl_1, NbStl_2, and NbStl_3; uninfected groups: CtlStl_1, CtlStl_2, and CtlStl_3). (*n* = 5 individual silkworms, samples were combined and analyses were done in triplicate).

Next, the effects of *N. bombycis* infection on gut eukaryotes were investigated by ITS sequencing analysis. Shannon and Chao1 index analysis confirmed that *N. bombycis* infection significantly down-regulated the eukaryotes diversity in digestive juice and stools (Fig. 5A). Principal coordinates analysis (PCoA) demonstrated that *N. bombycis* was able to cause the separation of microbial clusters from the control group, indicating that *N. bombycis* infection interfered with the structure of the intestinal microbiota of silkworms (Fig. 5B). We further analyzed the species compositions of the two samples and found that *N. bombycis* infection led to dramatic disturbance of eukaryote compositions. In the digestive juice samples, the *Penicillium*, *Trichomeriaceae_gen_Incertae_sedis*, *Chaetasbolisia, Moesziomyces,* etc., were upregulated, while *Aspergillus*, *Humicola*, *Stachybotrys,* etc., were downregulated after *N. bombycis* infection (Fig. 5C). As for the microorganisms in the stool samples, our analysis indicated that *Cladosporium*, *Alternaria, Ustilag,* etc., were upregulated, while *Nigrospora*, *Periconia, Botrytis,* etc., were downregulated after *N. bombycis* infection (Fig. 5D). Funguild was also applied to analyze the functional annotation database of fungi. We found that infection resulted in significant upregulation of plant pathogen (pathotroph), animal pathogen-plant pathogen-undefined saprotroph (pathotroph-saprotroph), and epiphyte (symbiotroph) in digestive juice samples, suggesting that the fungal community in digestive juice was mainly pathotroph (Fig. 6A). For the stool samples, the animal pathogen-endophyte-lichen parasite-plant pathogen-wood saprotroph (pathotroph-symbiotroph-saprotroph) and animal pathogen-endophyte-plant pathogen-wood saprotroph (pathotroph-symbiotroph-saprotroph) were upregulated, suggesting that the nutrient type of fungal community in silkworm sand was mainly pathotroph-symbiotroph-saprotroph mixed type (Fig. 6B). Taken together, our findings demonstrated that *N. bombycis* infection fundamentally changed the composition and function of the host gut prokaryotic and eukaryotic microorganisms.

### *N. bombycis* infection impaired the host digestive enzymes and capability

**Fig 7:**
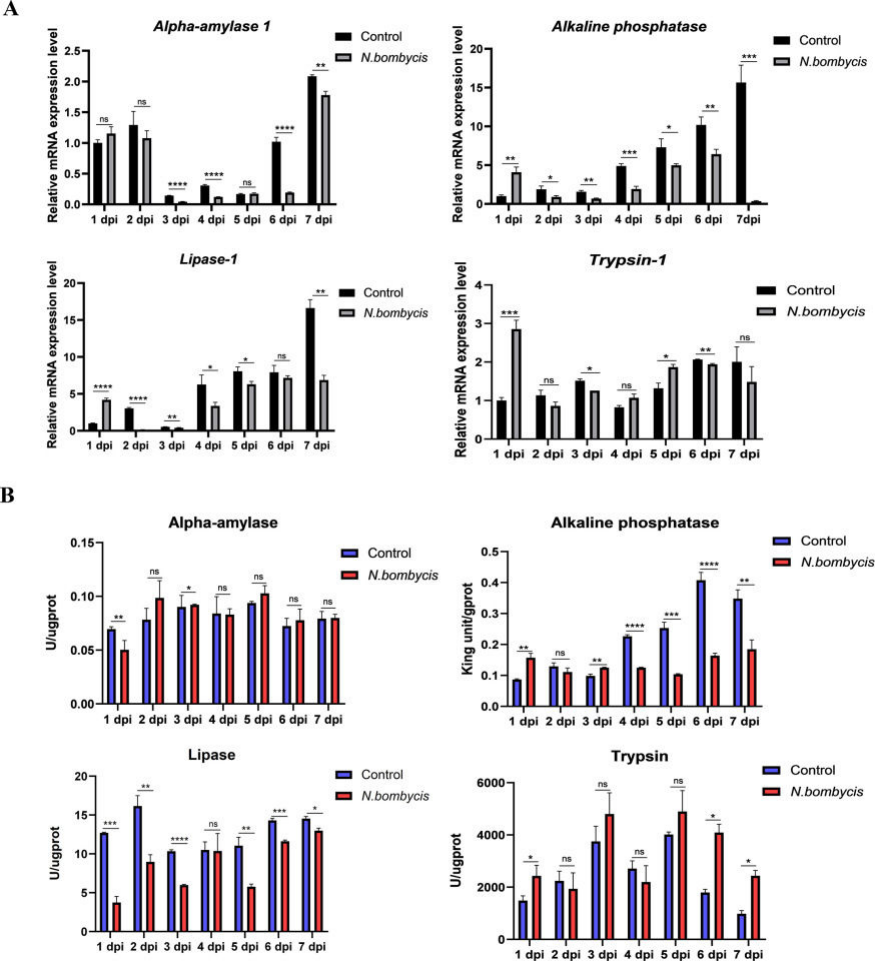
Expression levels and enzyme activities of *B. mori* digestive enzymes. (**A**) *Alpha-amylase 1*, *Alkaline phosphatase*, *Lipase-1*, and *Trypsin 1* expressions were detected after *N. bombycis* infection by qPCR. The expressions of *Alpha-amylase 1*, *Alkaline phosphatase,* and *Lipase-1* were significantly decreased; *Trypsin 1* was downregulated in 3 dpi while upregulated in 4 dpi, 5 dpi, and 6 dpi (*n* = 5; ns = not significant, *P < 0.05, **P < 0.01, ***P < 0.001, ****P < 0.0001). (**B**) Enzyme activities were detected after *N. bombycis* infection in the digestive juice. The enzyme activities of alpha-amylase were downregulated in 1 dpi, with no significant differences since; Activities of alkaline phosphatase and lipase were downregulated from 1 dpi to 7 dpi. Trypsin activity was upregulated significantly on 1 dpi, 6 dpi, and 7 dpi (*n* = 5; ns = not significant, *P < 0.05, **P < 0.01, ***P < 0.001, ****P < 0.0001).

Silkworms showed indigestion after *N. bombycis* infection. Therefore, we investigated the expression levels and enzyme activities of various digestive enzymes upon *N. bombycis* infection. qPCR analysis proved that the expressions of *Alpha-amylase 1, Alkaline phosphatase,* and *Lipase-1* were downregulated after infection; only *Trypsin 1* was upregulated in the later stages of infection (Fig. 7A). Enzyme activity measurements showed that the enzyme activity of alpha-amylase was downregulated on 1 dpi, and no significant change since; while the alkaline phosphatase and lipase activities were significantly reduced, and the trypsin activity was increased in the later stage of infection (Fig. 7B). These findings further confirmed the impaired effects of *N. bombycis* on host digestive capability and explain the indigestion and growth retardation of *N. bombycis*-infected silkworms.

### BmCDA8 possessed a key protective function against *N. bombycis* infection

**Fig 8:**
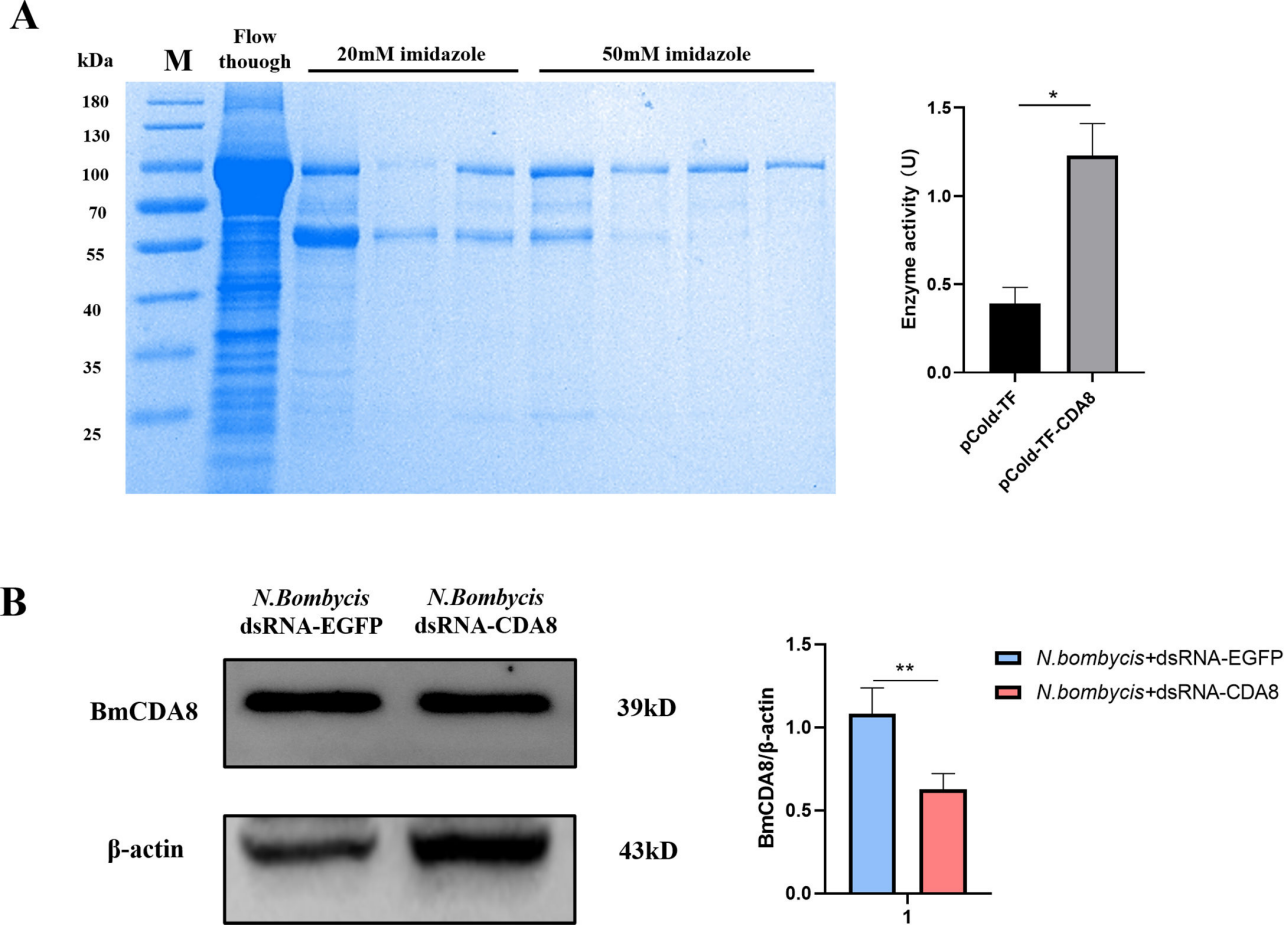
BmCDA8 purification, enzymatic activity, and RNAi effect analysis. (**A**) Recombinant BmCDA8 was purified, and its deacetylase activity was confirmed. Deacetylase activity was detected by the 4-nitroacetanilide method. Control was treated with pCold-TF (*P < 0.05). (**B**) The effect on the protein levels after BmCDA8 interference and *N. bombycis* infection. Densitometric quantification of the ratios of total BmCDA8 to β-actin from Western blot signals is shown (*n* = 3 experiments, **P < 0.01). Control was treated with dsRNA*-EGFP*.

**Fig 9:**
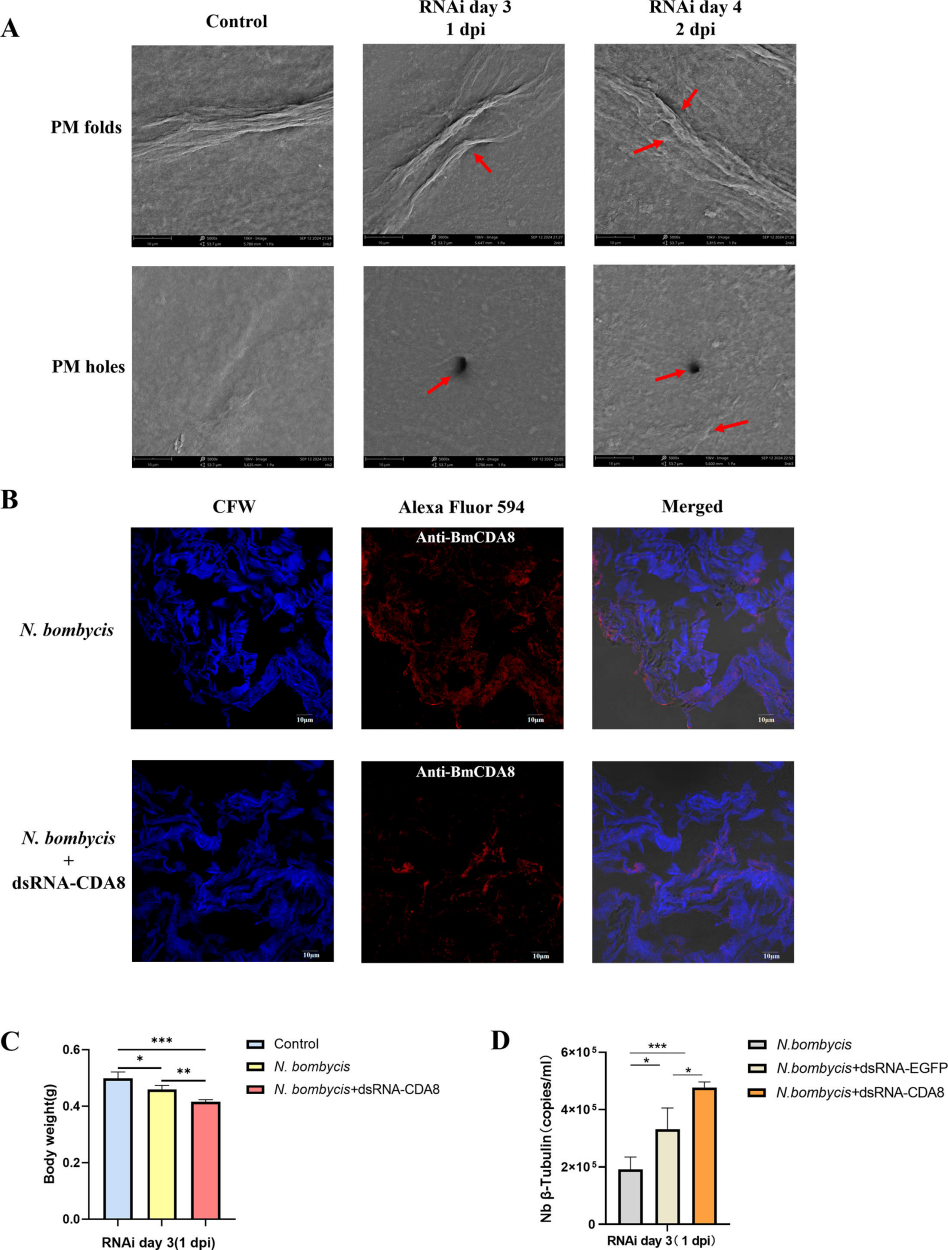
Key role of BmCDA8 in protective function and *N. bombycis* infection. (**A**) SEM observation of the peritrophic membrane after knockdown of the expression of BmCDA8. Significantly more anomalous folds and holes (red arrows) after being treated with dsRNA*-BmCDA8* (magnification, 5,000×). (**B**) Peritrophic membrane was stained with calcofluor white (CFW, Fluorescent brightener 28) (blue) and anti-BmCDA8 (Alexa Fluor 594, red). The peritrophic membrane in the *N. bombycis* + dsRNA-CDA8 treated group appeared looser compared to the *N. bombycis*-infected group (scale bar: 10 µm). (**C**) Body weight measurement after BmCDA8 interference and *N. bombycis* infection. The body weight of the *N. bombycis* + dsRNA-BmCDA8 treated group was significantly lower than the control group and *N. bombycis*-infected group (*P < 0.05, **P < 0.01, ***P < 0.001). (**D**) *N. bombycis* loads were determined by qPCR. The number of *N. bombycis* was significantly higher in the dsRNA*-BmCDA8-*treated group, compared to *N. bombycis* infection only and dsRNA-*EGFP*-treated groups (*P < 0.05, ***P < 0.001).

**Fig 10:**
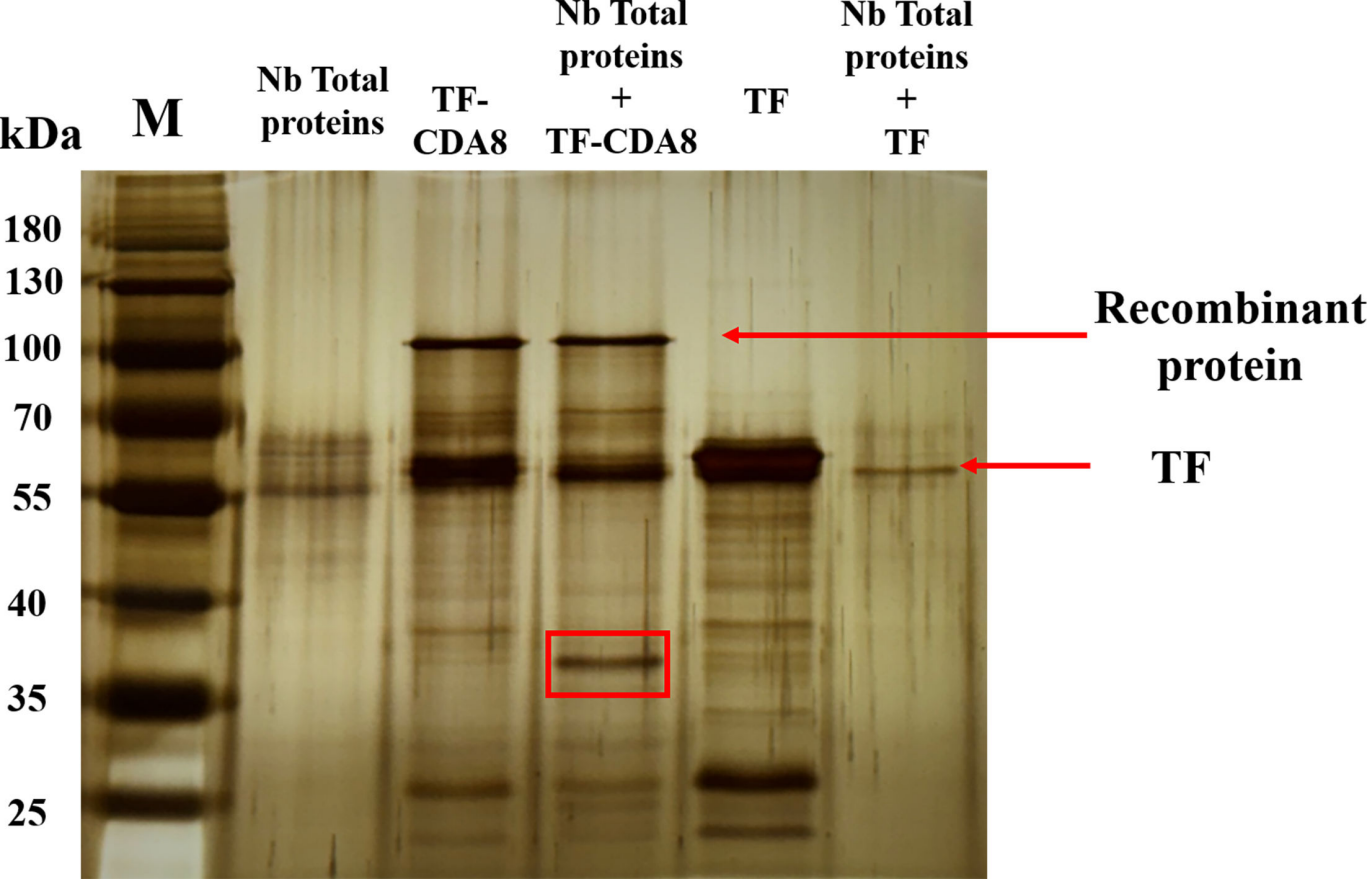
Using recombinant pCold-TF-BmCDA8 protein as bait, total proteins from *N. bombycis* were incubated for His pull-down assay, followed by SDS-PAGE and silver staining of eluted proteins. The differential band was highlighted with a red box, representing the potential interacting proteins. The control group was treated with pCold-TF empty plasmid.

**Fig 11:**
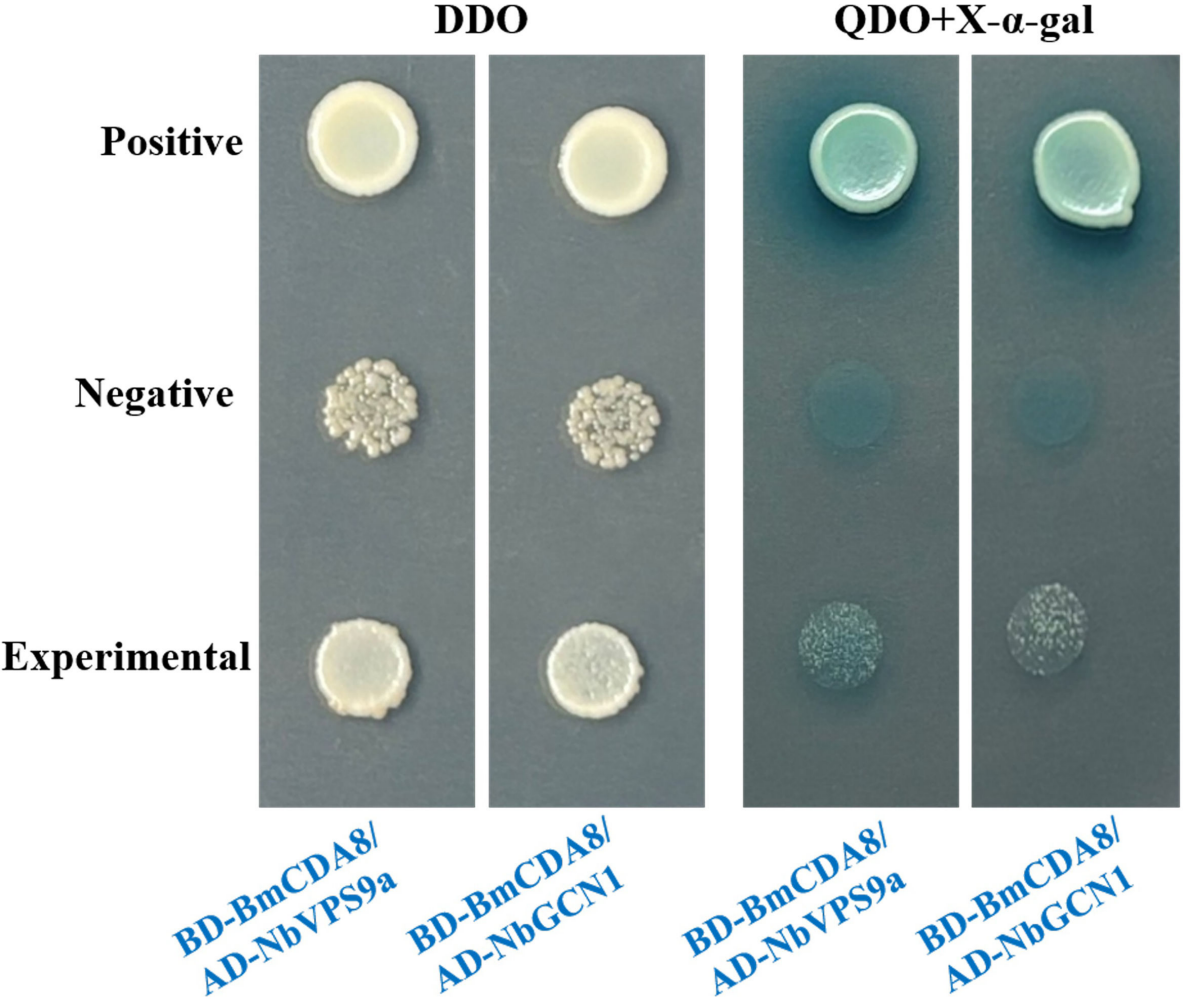
Yeast two-hybrid analysis of interactions among BmCDA8 and NbVPS9a, NbGCN1. DDO, SD/-Leu/-Trp; QDO, SD/-Ade/-His/-Leu/-Trp.

Chitin deacetylases (CDAs) are expressed by the midgut and are responsible for trimming the major building block, chitin, on the peritrophic membrane. In addition to accumulating evidence to show the involvement of CDAs during host-pathogen interactions, we also noticed from our analysis that BmCDA8 was among the top dysregulated proteins after *N. bombycis* infection. Therefore, we purified recombinant BmCDA8 and confirmed its deacetylase activity (Fig. 8A). Next, we validated whether BmCDA8 is essential for membrane integrity. Since the peritrophic membrane is the entry port of *N. bombycis* into the host, we inferred that downregulation of BmCDA8 leads to a more fragile membrane and subsequent more infection of the pathogen. RNA interference (RNAi) assay was applied to knock down the expression of BmCDA8 by injecting BmCDA8-dsRNA (Fig. 8B). SEM showed that the surface of the peritrophic membrane in the BmCDA8 group had more folds and holes than the *N. bombycis* infection group (Fig. 9A). Immunofluorescence analysis revealed that interference with BmCDA8 expression leads to more distorted peritrophic membrane structure (Fig. 9B). Compared to the *N. bombycis*-infected only controls, the silkworm group that was interfered with BmCDA8 expression and infected by *N. bombycis* showed more weight loss (Fig. 9C) and increased the pathogen loads (Fig. 9D). To screen and identify *N. bombycis* effectors that potentially interact with and affect BmCDA8, His pull-down assays were applied using the recombinant BmCDA8 as bait. By mass spectrometry analysis of the pulled-down proteins, we identified 24 *N*. *bombycis* proteins (Fig. 10, Table 6). Coordination analysis of these proteins and the *N. bombycis* proteins we identified in the midgut (Table 5), we infer that ubiquitin (fragment), vacuolar protein sorting-associated protein 9a, GCN1-like translational activator, and Tail completion protein S may be the effectors that interact with BmCDA8. Subsequently, we employed the yeast two-hybrid system to elucidate the interactions between BmCDA8 and the candidate target proteins. We demonstrated that *N. bombycis*-derived vacuolar protein sorting-associated protein 9a (NbVPS9a) and GCN1-like translational activator (NbGCN1) interact with BmCDA8 directly (Fig. 11).

**TABLE 6:** His–BmCDA8 pulled down 24 proteins from total proteins of *N. bombycis*

Protein	Accession number	Molecular weight (kDa)
40S ribosomal protein S25	R0KXE1; F2 X0Z3	7.8621
Ubiquitin (fragment); 40S ribosomal protein S31 (fragment)	R0KPY3; F2X108	15.818
Vacuolar proton pump subunit d 2; V-type proton ATPase subunit	R0ML21; Q15EY5	40.259
CDT1 geminin-binding domain-containing protein	R0KLY5	51.604
Uncharacterized protein	R0KN04	16.077
Actin (fragment)	R0KN67; R0MJB1; R0M8Z2	18.109
Dynamin-2; uncharacterized protein	R0MCB7; R0KNF6	20.539
Vacuolar sorting protein 9; vacuolar protein sorting-associated protein 9a	R0MFA0; R0KSW6	25.63
Uncharacterized protein	R0KT40	16.89
tRNA pseudouridine synthase D	R0KTC0	27.206
GCN1-like translational activator; GCN1-like translational activator (fragment)	R0MFR0; R0KUJ0	13.239
Uncharacterized protein	R0KWK5	21.689
Uncharacterized protein	R0MFI3; R0KXY5	8.3577
Vacuolar proton pump subunit D	R0KZ58	24.962
General transcription factor IIF subunit 2	R0M2W3	26.258
Kinesin-2	R0M6A3	55.096
Uncharacterized protein	R0MM41; R0M6Y2	31.532
Target SNARE coiled-coil region	R0M916	10.528
Uncharacterized protein	R0MF04	39.461
Tail completion protein S	R0MFQ0	8.0984
High affinity cAMP-specific and IBMX-insensitive 3,5-cyclic phosphodiesterase 8A	R0MGU3	34.118
Uncharacterized protein	R0MH05	19.601
DNA-directed RNA polymerase I subunit RPA12	R0MJ59	11.143
Uncharacterized protein	R0MNI3	47.323

## DISCUSSION

Silkworm midgut, including the intestinal epithelium and peritrophic membrane, is an essential barrier for protection, digestion, and absorption (25–27). A comprehensive investigation of the effects of *N. bombycis* on the silkworm intestinal barrier would be of great importance for disease prevention and control. In this study, we utilized various methods to investigate the three aspects of barrier integrity and function changes upon *N. bombycis* infections. SEM observations and permeability assay showed that the infection caused structural disruption and increased the permeability. A quantitative proteomics study revealed that midgut proteins were dysregulated after infection. GO analysis and KEGG pathway enrichment prediction analysis indicated these proteins/related genes were associated with membrane function/structure, catalytic activity, and digestive functions. 16S rRNA and ITS analysis of digestive juice and stool samples revealed that host gut microbiome profiles were significantly disturbed after infection. Digestive enzyme expressions and their activities were also disrupted after *N. bombycis* infection. These results demonstrated a comprehensive image of the impaired effects of *N. bombycis* on host intestinal barrier integrity and function.

The gut microbiota of silkworms assembles into increasingly identical communities throughout development, highlighting the effects of host specificity on microbial associations and the potential role these communities play in host biology (28, 29). Comparative analysis of silkworm intestinal microbiota and fungal microbiota showed that the biodiversity of intestinal microbiota decreased significantly, and the abundance and function of silkworm microbiota decreased significantly, which may be related to the intestinal health of the host (30, 31).

Gut commensal microorganisms, including both prokaryotes and eukaryotes, are sculptors of various biological functions. Our study demonstrated the dramatic distortion effects of *N. bombycis* on the host gut microorganism diversity, species composition, and functions. For example, we found that various types of microorganisms functioning in digestion or nutrient uptake were down-regulated after infection, such as *Curtobacterium* and *Pseudomonas* (32, 33). Some genera associated with beneficial and relieving effects were decreased, such as *Enterococcus,* and have been found to be beneficial bacteria in several biological models, which can reduce the invasion of pathogens and improve fish and bee health (34–36). In addition, some fungal microorganisms were significantly enriched after microsporidia infection, such as the saprophyte *Penicillium* and the plant pathogen *Moesziomyceus*. These fungi groups were also related to nutrition uptake modes (37, 38). The changes in both domains of microorganisms after infection indicate that *N. bombycis* profoundly affects the way of nutrition digestion and uptake in the host gut, therefore helping to explain the phenomena of ingestion and growth retardation after infection. Interestingly, we also noticed several differences related to microbiota composition between digestive juice and stool samples. For example, the genera of *Acinetobacter*, *Cutibacterium*, *Bacillus*, and *Microbacterium* were upregulated in the digestive juice but were downregulated in stool samples of *N. bombycis*-infected groups. Conversely, *Staphylococcus* and *Enterococcus* exhibited decreased abundance in digestive juice but were enriched in stool samples. As for the fungal communities, the variances and divergences between the two kinds of samples were even more obvious, with nearly totally different fungal groups in each sample.

To digest dietary proteins, carbohydrates, and lipases, insects also rely on the hydrolysis of gut digestive enzymes (39). Alpha-amylase mediates the initial digestion of carbohydrates, lipase is a key enzyme involved in lipid digestion, and serine proteases (including trypsin) account for 95% of the total proteolytic activity in most insects’ guts (40–43). In our study, we found that the expressions of alpha-amylase, lipase-1, and alkaline phosphatase were downregulated after *N. bombycis* infection, while trypsin was upregulated. The changes in the activities of these digestive enzymes followed the same pattern. These results indicate that the silkworm’s intestinal chemical barrier and digestive capabilities were weakened by *N. bomycis*. Interestingly, trypsin is not only a digestive enzyme but also functions as a protease inhibitor and antimicrobial protein in silkworm pupae and cocoons (44). Therefore, the upregulation of trypsin after *N. bombycis,* as we found in our study, may reflect the fierce battle between host and pathogen.

Details and mechanisms about how *N. bombycis* passes through the *B. mori* midgut are still unknown (45). Our study by SEM provides the first-hand and crystal-clear evidence to show that *N. bombycis* would make distortions and holes on the peritrophic membrane as well as the midgut epithelial membrane to facilitate penetration through the barriers. Our proteomics and RNAi analysis further pointed out the key roles of a chitin deacetylase (CDA), BmCDA8, during the distortion of the membrane. CDAs are known for the ability to degrade chitin into chitosan, which mainly plays a role in removing part of the acetylglucosamine residues and modifying chitin in the process of peritrophic membrane renewal and formation (8, 46). A previous study reported that CDAs could also mediate pathogen infection and related barrier disruption. When baculovirus-infected cotton bollworm larvae, the midgut HaCDA5a gene was downregulated, and the permeability of the recombinant protein HaCDA5a was increased after incubation with *Bactrocera dorsalis* peritrophic membrane, indicating that HaCDA5a was involved in changing peritrophic membrane permeability and was related to the invasion of pathogens (47). In our study, BmCDA8 was upregulated after *N. bombycis* infection, the permeability of the membrane was increased after incubation of recombinant BmCDA8, and the pathogen load would be reduced after knocking down BmCDA8. These findings supported our hypothesis that BmCDA8 is the key modulator of membrane integrity and the main target of *N. bombycis* invasion, therefore leading to the distortions and impairments of gut integrity and function. The vacuolar protein sorting-associated protein typically functions as a guanine nucleotide exchange factor (GEF), activating Rab GTPases to regulate vesicular trafficking and membrane fusion (48, 49). The GCN1-like translational activator acts as a critical translational regulator in protein homeostasis, serving as a key component of translational quality control systems (50). Meanwhile, chitin deacetylases (CDAs) catalyze the deacetylation of chitin to generate chitosan, which may mediate biofilm interactions. Based on the above, we therefore hypothesize that during *N. bombycis* infection of the host intestinal tract, pathogen proteins NbVPS9a and NbGCN1 interact with host BmCDA8 via dual mechanisms. NbVPS9a mediates cytosolic membrane fusion, which may lead to dysregulation or upregulation of BmCDA8 secretion and peritrophic membrane chitin metabolism. NbGCN1 in the cytosol may also target the translational regulation of BmCDA8, which could dysregulate chitin synthesis and remodeling within the peritrophic membrane.

The physical barrier plays a pivotal role in initial pathogen exclusion. BmCDA8 regulates the synthesis and modification of chitin in the peritrophic membrane, thereby maintaining the integrity of the intestinal physical barrier. The loss of physical barrier integrity can trigger severe physiological effects: interfering with the chemical barrier composed of digestive enzymes and immune factors, disrupting intestinal microbial homeostasis (51, 52). Gut barrier imbalance ultimately weakens the host’s resistance to pathogens and leads to metabolic disorders and the development of various diseases (53). BmCDA8 helps maintain barrier integrity, thereby ensuring the stable expression and functional activity of digestive enzymes, as well as the homeostasis of microbial colonization in the gut. Therefore, alterations in BmCDA8 may affect these three aspects of the gut barrier. To verify the changes of microbiota after RNAi, we tested the representative *Enterococcus* genera, which downregulated in digestive juice while upregulated in stool after *N. bombycis* infection. After BmCDA8 expression was interfered, the *Enterococcus* genera dysregulated in both digestive juice and stool sample compared to *N. bombycis* infection alone. The other representative bacteria genera is *Bacillus* genera, which is upregulated in digestive juice while downregulated in stool after *N. bombycis* infection. After BmCDA8 expression was interfered, the compositions reverted in either sample. The above results confirmed that change of BmCDA8 did affect the microbiota composition. These results are now shown in Fig. S2. In addition, the expression changes in the digestive enzymes after BmCDA8 expression were interfered. Compared with *N. bombycis* infection alone, the expression of *Alpha-amylase 1* and *Lipase-1* was downregulated, while *Alkaline phosphatase* and *Trypsin 1* were upregulated (Fig. S3). Therefore, alterations in BmCDA8 can influence the homeostasis of the gut’s physical barrier, microbiota barrier, and chemical barrier.

## Data Availability

The full set of gut microbiome data about bacteria and fungi metagenome raw sequence reads has been deposited into the Genome Sequence Archive (GSA) of the National Genomics Data Center of Chinese Academy of Sciences (accession number CRA026568). The silkworm midgut proteomic data and the pull-down proteins’ proteomic data have been deposited into the OMIX of the National Genomics Data Center of Chinese Academy of Sciences (accession numbers OMIX010492 and OMIX010493). All information is freely open and accessible.

## SUPPLEMENTAL MATERIAL

Figure S1 msphere.00095-25-s0001.tif Synthesis of dsRNA-EGFP and dsRNA-BmCDA8 interference fragments.

Figure S2 msphere.00095-25-s0002.tif PCR analysis of representative gut microbiota species.

Figure S3 msphere.00095-25-s0003.tif Digestive enzymes expressions.

Legends msphere.00095-25-s0004.docx Supplemental figure legends.

Table S1 msphere.00095-25-s0005.docx Primer sets.

ASM does not own the copyrights to Supplemental Material that may be linked to, or accessed through, an article. The authors have granted ASM a non-exclusive, world-wide license to publish the Supplemental Material files. Please contact the corresponding author directly for reuse.
