# Supplementary figures and images for: Comprehensive characterization of the impairing effects of Nosema bombycis on the host digestive integrity and function
Source: mSphere. 2025 Jul 22;10(8):e00095-25. doi: 10.1128/msphere.00095-25 (PMC12379596; doi:10.1128/msphere.00095-25)

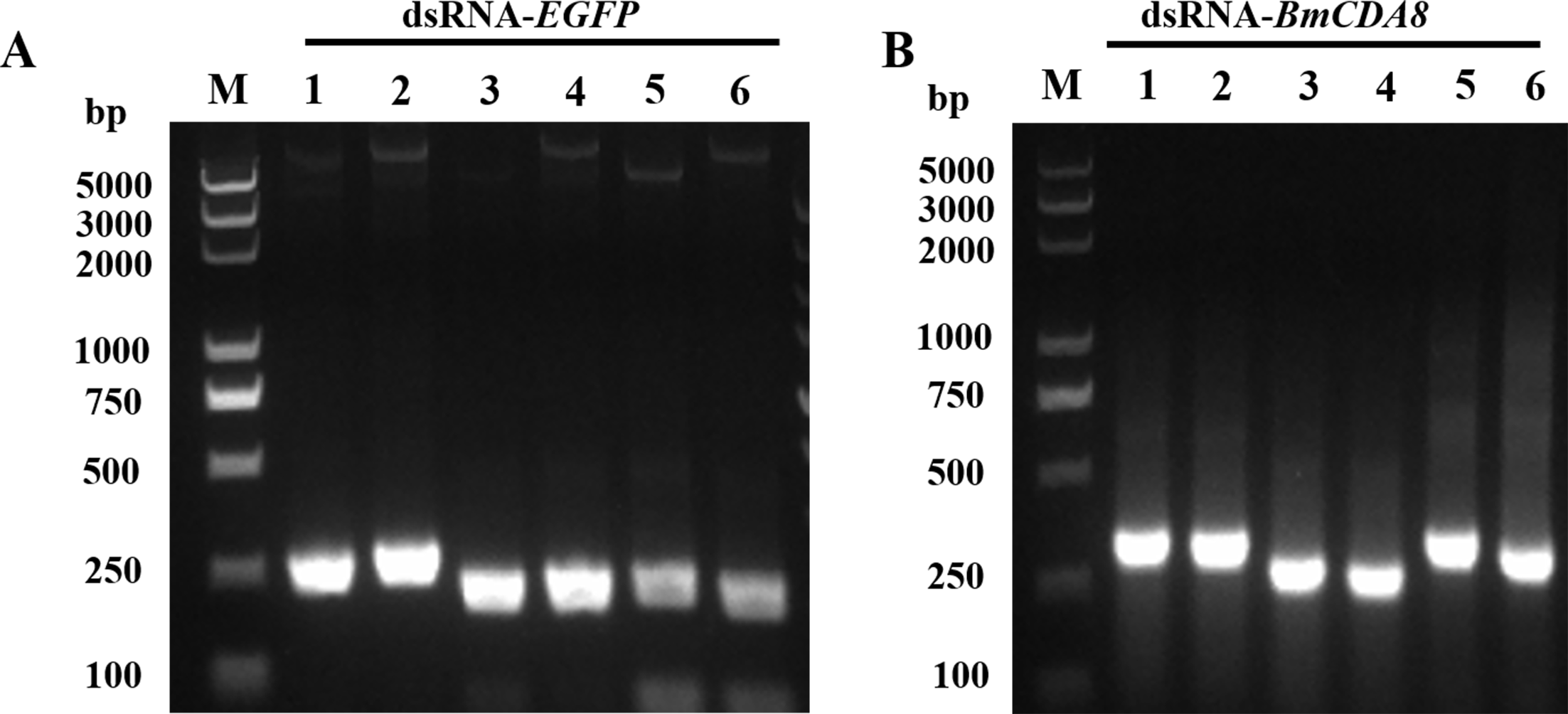

Supplement: Figure S1 — Synthesis of dsRNA-EGFP and dsRNA-BmCDA8 interference fragments. [file msphere.00095-25-s0001.tif]

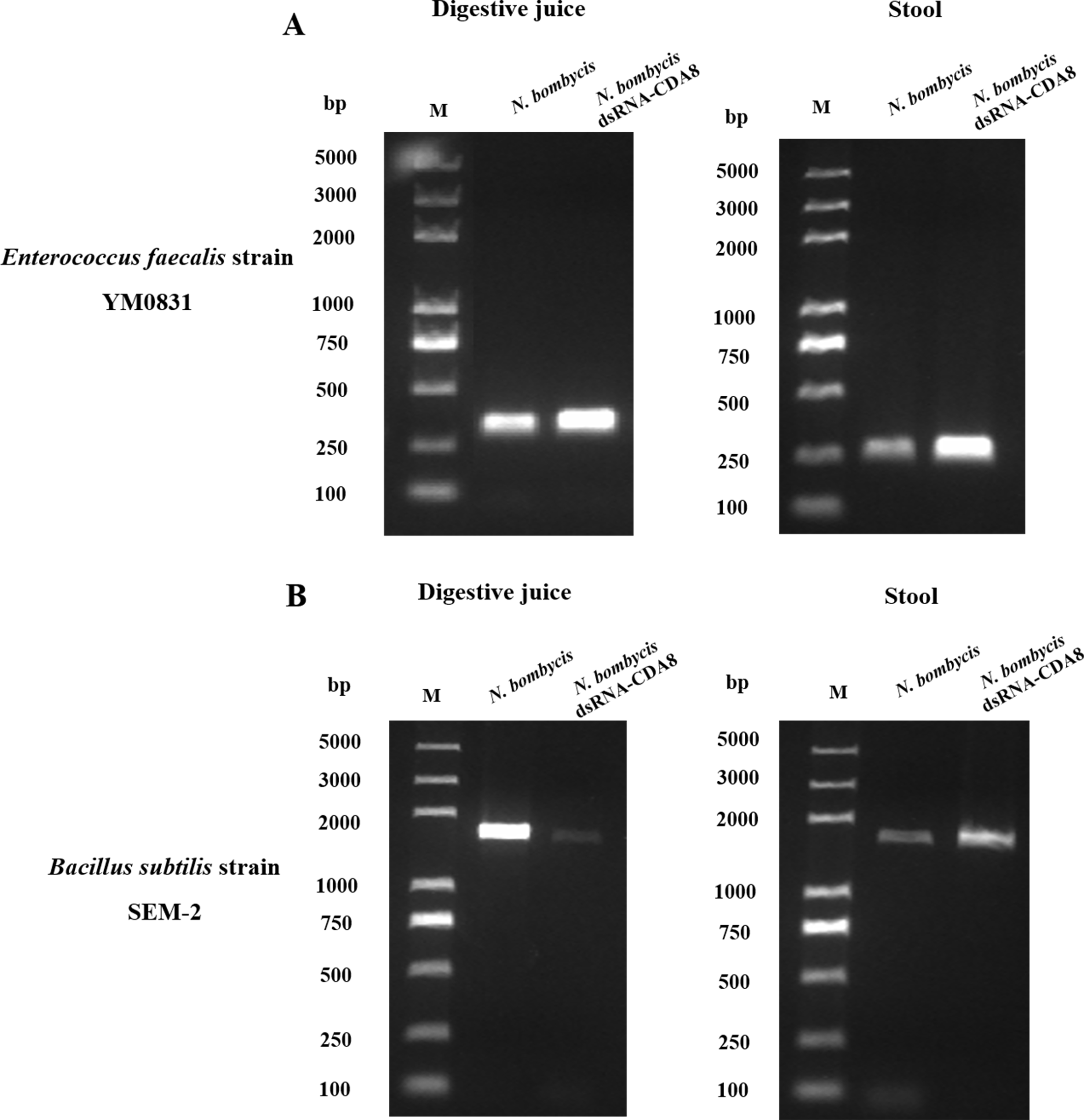

Supplement: Figure S2 — PCR analysis of representative gut microbiota species. [file msphere.00095-25-s0002.tif]

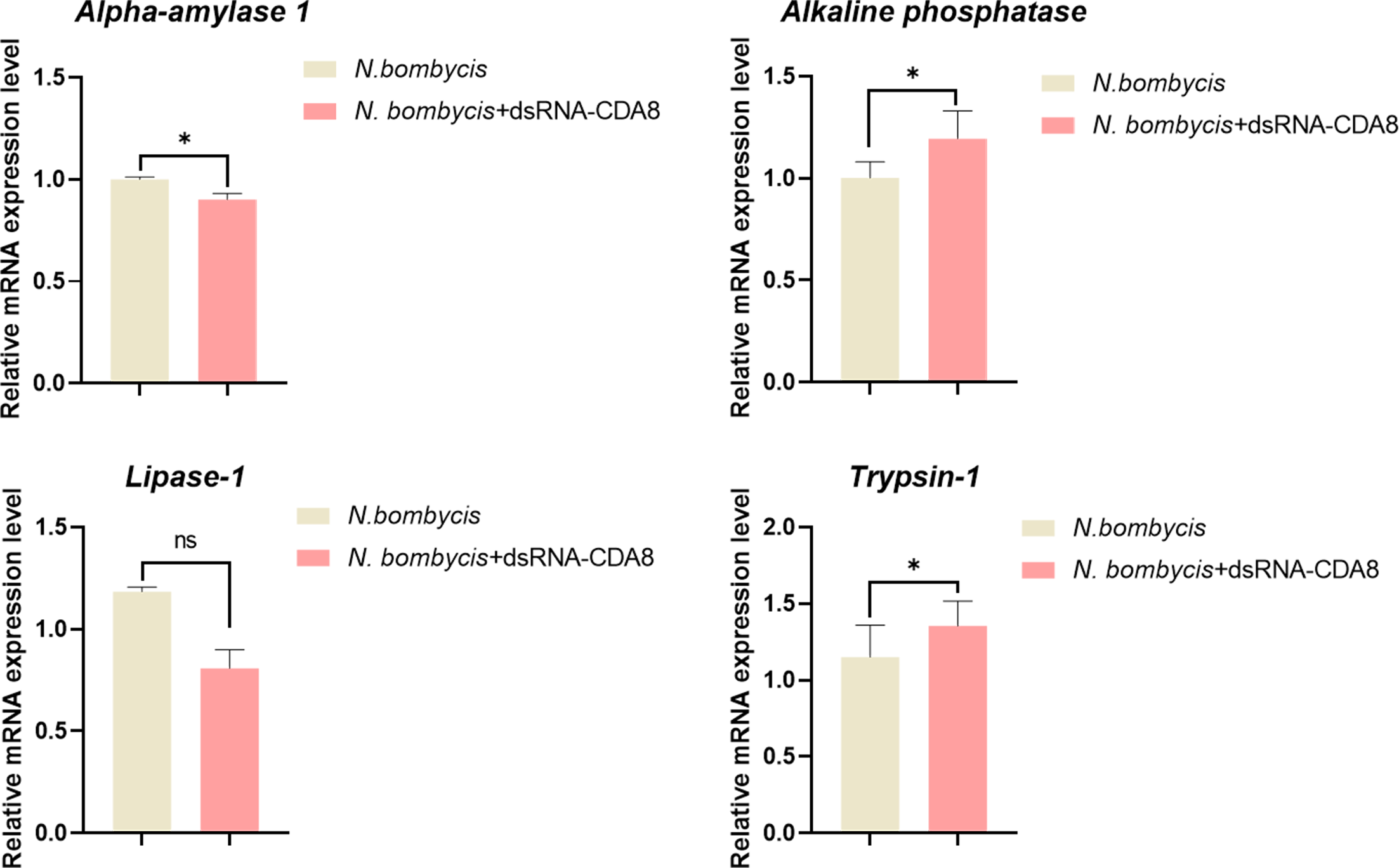

Supplement: Figure S3 — Digestive enzymes expressions. [file msphere.00095-25-s0003.tif]
