# Supplementary material for: Comprehensive characterization of the impairing effects of Nosema bombycis on the host digestive integrity and function
Source: mSphere. 2025 Jul 22;10(8):e00095-25. doi: 10.1128/msphere.00095-25 (PMC12379596; doi:10.1128/msphere.00095-25)
Supplement: Legends — Supplemental figure legends. [file msphere.00095-25-s0004.docx]

**Fig. S1.** Synthesis of dsRNA-EGFP and dsRNA-BmCDA8 interference fragments. M: 2K Plus DNA Marker; A1-2: T7EGFP-1 interference fragment; A3-4: T7EGFP-2 interference fragment; A5-6: T7EGFP-3 interference fragment; B1-2: T7BmCDA8-1 interference fragment; B3-4: T7BmCDA8-2 interference fragment; B5-6: T7BmCDA8-3 interference fragment.

**Fig. S2** PCR analysis was conducted to evaluate alterations in representative gut microbiota species (RNAi day 3, 1 dpi). **(A)** *Enterococcus faecalis* strain YM0831, genomic DNA standardized to 50 ng per digestive juice and stool samples. **(B)** *Bacillus subtilis* strain SEM-2, genomic DNA standardized to 50 ng per digestive juice and stool samples.

**Fig. S3** Digestive enzymes expressions were detected after BmCDA8 expression was inhibited by RNAi by qPCR (RNAi day 3, 1 dpi). The expressions of *Alpha-amylase 1*, *Lipase-1* were decreased; *Alkaline phosphatase* and *Trypsin 1* were up-regulated (n=5. ns=not significant, *= P<0.05).
