# Supplementary material for: Comprehensive characterization of the impairing effects of Nosema bombycis on the host digestive integrity and function
Source: mSphere. 2025 Jul 22;10(8):e00095-25. doi: 10.1128/msphere.00095-25 (PMC12379596; doi:10.1128/msphere.00095-25)
Supplement: Table S1 — Primer sets. [file msphere.00095-25-s0005.docx]

**Table S1** Primers used in this study.

| Purpose | Construct | Primer sequences | |
| --- | --- | --- | --- |
|  |  | Forward | Reverse |
| RT-qPCR assay | q- alpha-amylase 1 | 5'- GCTGCTGAATGCGAAAGGT -3' | 5'- GGTAGGAGATTGGTTGATAGCG -3' |
|  | q- trypsin-1 | 5'- CGCATCCAGAGTATGACAACCC -3' | 5'- TGACCGATACCTCCGTTCCA-3' |
|  | q- lipase-1 | 5'- TGGAACAGCAACGGAAACTC -3' | 5'- CACTGGGAACTCCATTGACG -3' |
|  | q- alkaline phosphatase | 5'- GTAACGACCGACGCCAACTT -3' | 5'- ATGCCGCAAGGTGATGAAG -3' |
|  | q-Nb-β-tubulin | 5'-AGAACCAGGAACAATGGACG-3' | 5'- AGCCCAATTATTACCAGCACC-3' |
| For construction of expression vector | pCold-TF-BmCDA8 | 5'- GAGCTCGGTACCCTCGAGGGATCCCGAGCCAGAGACACTCCACAG -3' | 5'- CTAGACTGCAGGTCGACAAGCTTCTGTCCAAGCGGATTTCCTACC -3' |
| dsRNA assay | T7EGFP-1  T7EGFP-2  T7EGFP-3  T7BmCDA8-1  T7BmCDA8-2  T7BmCDA8-3 | 5'- TAATACGACTCACTATAGGGAGAACGGCAAGCTGACCCTGAAGTT -3'  5'-TAATACGACTCACTATAGGGAGACACATGAAGCAGCACGACT-3'  5'-TAATACGACTCACTATAGGGAGACCATCTTCTTCAAGGACGA-3'  5'-TAATACGACTCACTATAGGGAGACCGGTAACACAAGTTTCCAG-3'  5'-TAATACGACTCACTATAGGGAGACGAATCTATTCAGGACTGCAT-3  5'-TAATACGACTCACTATAGGGAGAGACGAGGAAGGATGGTTCAAGT-3' | 5'-TAATACGACTCACTATAGGGAGATTGAAGTCGATGCCCTTCAGCTCGA-3'  5'-TAATACGACTCACTATAGGGAGAATGATATAGACGTTGTGGCTGTT-3'  5'-TAATACGACTCACTATAGGGAGATTGTGGCGGATCTTGAAGT-3'  5'-TAATACGACTCACTATAGGGAGATAAACTTGAACCATCCTTCCTCGTC-3'  5'-TAATACGACTCACTATAGGGAGAATTCGTGGACGAAGAATCCGAACGG-3'  5'-TAATACGACTCACTATAGGGAGATAAGACAGCTGATTATGAGTTCCCG-3' |
| Yeast two-hybrid assay | pGBKT7-BmCDA8  pGADT7-NbVPS9a  pGADT7-NbGCN1 | 5'-TCAGAGGAGGACCTGCATATGCGAGCCAGAGACACTCCACAG-3'  5'-GCCATGGAGGCCAGTGAATTCATGAAACCATCACTTCCCAGG-3'  5'-ATGGAGGCCAGTGAATTCATGTGTGATACTAAAATACAGAGA-3' | 5'-CGACGGATCCCCGGGAATTCCTGTCCAAGCGGATTTCCTACC-3'  5'-CAGCTCGAGCTCGATGGATCCCCATCATTTTAAGATCAAACT-3'  5'-GCTCGAGCTCGATGGATCCCAGATAAAAATACAAGCAAGTT-3' |
| PCR detect gut microbes | YM0831 | 5'-GGAGGCAGCAGTAGGGAATCTT-3' | 5'-GCTCGCTTTACGCCCAATA-3' |
|  | SEM-2 | 5'-TTGGAGACTTTACTGGTTGT-3' | 5'-TTACGCTTCTTCAGCTTCAATCACT-3' |
